# Differential association of GABA_B_ receptors with their effector ion channels in Purkinje cells

**DOI:** 10.1007/s00429-017-1568-y

**Published:** 2017-11-25

**Authors:** Rafael Luján, Carolina Aguado, Francisco Ciruela, Javier Cózar, David Kleindienst, Luis de la Ossa, Bernhard Bettler, Kevin Wickman, Masahiko Watanabe, Ryuichi Shigemoto, Yugo Fukazawa

**Affiliations:** 1 Departamento de Ciencias Médicas, Facultad de Medicina, Instituto de Investigación en Discapacidades Neurológicas (IDINE), Universidad Castilla-La Mancha, Campus Biosanitario, C/Almansa 14, 02006 Albacete, Spain; 2 Unitat de Farmacologia, Departament de Patologia i Terapèutica Experimental, Facultat de Medicina, IDIBELL, Universitat de Barcelona, 08907 L’Hospitalet de Llobregat, Spain; 3 Institut de Neurociències, Universitat de Barcelona, Barcelona, Spain; 4 Departamento de Sistemas Informáticos, Escuela Superior de Ingeniería Informática, Universidad de Castilla-La Mancha, 02071 Albacete, Spain; 5 Institute of Science and Technology Austria (IST Austria), 3400 Klosterneuburg, Austria; 6 Department of Biomedicine, Institute of Physiology, University of Basel, 4056 Basel, Switzerland; 7 Department of Pharmacology, University of Minnesota, 321 Church Street South East, Minneapolis, MN 55455 USA; 8 Department of Anatomy, Hokkaido University School of Medicine, Sapporo, 060-8638 Japan; 9 Department of Histological and Physiological Sciences, Faculty of Medical Science, University of Fukui, Yoshida, Fukui 910-1193 Japan

**Keywords:** Electron microscopy, Cerebellum, GABA_B_ receptors, Potassium channels, Calcium channels, Purkinje cells, Freeze-fracture replica immunolabelling, Synapses, Quantification, Parallel fibre, Active zone

## Abstract

Metabotropic GABA_B_ receptors mediate slow inhibitory effects presynaptically and postsynaptically through the modulation of different effector signalling pathways. Here, we analysed the distribution of GABA_B_ receptors using highly sensitive SDS-digested freeze-fracture replica labelling in mouse cerebellar Purkinje cells. Immunoreactivity for GABA_B1_ was observed on presynaptic and, more abundantly, on postsynaptic compartments, showing both scattered and clustered distribution patterns. Quantitative analysis of immunoparticles revealed a somato-dendritic gradient, with the density of immunoparticles increasing 26-fold from somata to dendritic spines. To understand the spatial relationship of GABA_B_ receptors with two key effector ion channels, the G protein-gated inwardly rectifying K^+^ (GIRK/Kir3) channel and the voltage-dependent Ca^2+^ channel, biochemical and immunohistochemical approaches were performed. Co-immunoprecipitation analysis demonstrated that GABA_B_ receptors co-assembled with GIRK and Ca_V_2.1 channels in the cerebellum. Using double-labelling immunoelectron microscopic techniques, co-clustering between GABA_B1_ and GIRK2 was detected in dendritic spines, whereas they were mainly segregated in the dendritic shafts. In contrast, co-clustering of GABA_B1_ and Ca_V_2.1 was detected in dendritic shafts but not spines. Presynaptically, although no significant co-clustering of GABA_B1_ and GIRK2 or Ca_V_2.1 channels was detected, inter-cluster distance for GABA_B1_ and GIRK2 was significantly smaller in the active zone than in the dendritic shafts, and that for GABA_B1_ and Ca_V_2.1 was significantly smaller in the active zone than in the dendritic shafts and spines. Thus, GABA_B_ receptors are associated with GIRK and Ca_V_2.1 channels in different subcellular compartments. These data provide a better framework for understanding the different roles played by GABA_B_ receptors and their effector ion channels in the cerebellar network.

## Introduction

GABA_B_ receptors are the G protein-coupled receptors for GABA, the main inhibitory neurotransmitter in the brain, and through coupling to different intracellular signal transduction mechanisms they mediate slow inhibitory postsynaptic potentials (IPSPs) (Bettler et al. 2004; Gassmann and Bettler 2012). Functional GABA_B_ receptors are obligate heterodimers composed of GABA_B1_ and GABA_B2_ subunits, and they are implicated in a number of disorders, including cognitive impairments, nociception, anxiety, depression and epilepsy (Bettler et al. 2004; Luján and Ciruela 2012; Luján et al. 2014). Depending on their subcellular localisation, GABA_B_ receptors exert distinct regulatory effects on synaptic transmission (Gassmann and Bettler 2012; Luján and Ciruela 2012). Stimulation of postsynaptic GABA_B_ receptors generally triggers inhibition of adenylate cyclase and activation of G protein-gated inwardly rectifying K^+^ (GIRK/Kir3) channels, leading to cell hyperpolarisation (Kaupmann et al. 1998). Presynaptic GABA_B_ receptors, however, suppress neurotransmitter release by depressing Ca^2+^ influx via P/Q-type and N-type voltage-gated Ca^2+^ (Ca_V_) channels (Huston et al. 1995; Takahashi et al. 1998; but see Zhang et al. 2016). There is now substantial evidence showing that GABA_B_ receptors, their cognate G proteins and downstream effectors are organised as macromolecular complexes (Clancy et al. 2005; David et al. 2006; Jaén and Doupnik 2006; Fowler et al. 2007; Fernández-Alacid et al. 2009; Ciruela et al. 2010; Laviv et al. 2011; Fajardo-Serrano et al. 2013; Schwenk et al. 2016). This data favours the idea that the spatial proximity of the interacting proteins seems to be a general mechanism to ensure that signalling is specific and fast.

In situ hybridization and immunohistochemical studies have shown that Purkinje cells (PCs), the output neurons of the cerebellar cortex, are the neuron type with the highest levels of GABA_B_ receptors (Bowery et al. 1987; Chu et al. 1990; Turgeon and Albin 1993; Kaupmann et al. 1997; Bischoff et al. 1999; Fritschy et al. 2004; Luján and Shigemoto 2006). Although electrophysiological and pharmacological studies have characterised pre- and postsynaptic inhibitory functions of GABA_B_ receptors in PCs (Batchelor and Garthwaite 1992; Dittman and Regehr 1996; Vigot and Batini 1997), the spatial relationship of GABA_B_ and their effector ion channels in various subcellular compartments of central neurons remains mostly unknown. Consistent with the functional coupling of GABA_B_ receptors with GIRK and Ca_V_ channels, immunohistochemical studies have shown that PCs have high density of GIRK channels (Aguado et al. 2008; Fernández-Alacid et al. 2009) and Ca_V_2.1 (P/Q-type) channels (Kulik et al. 2004; Indriati et al. 2013). These ion channels have been detected at postsynaptic sites along dendrites and spines of PCs, as well as presynaptically at parallel fibre terminals (Kulik et al. 2004; Aguado et al. 2008; Fernández-Alacid et al. 2009; Indriati et al. 2013).

To visualise the two-dimensional distribution of GABA_B_ receptors along the surface of PCs, as well as their spatial relationship with GIRK2 and Ca_V_2.1 channels, we used the freeze-fracture replica immunogold labelling (SDS-FRL) method, a highly sensitive and quantitative immunoelectron microscopic technique (Masugi-Tokita and Shigemoto 2007). This approach allowed us to examine the numbers, densities, and co-localization of these functionally coupled signalling proteins at post- and pre-synaptic membranes, allowing us to evaluate in a quantitative fashion the compartment-dependent association and segregation of GABA_B_ receptors and effector channels.

## Materials and methods

### Animals

Three adult C57BL/6J mice obtained from the Animal House Facility of the National Institute for Physiological Sciences (NIPS, Okazaki, Japan) were used in this study for immunoelectron microscopic analyses. For Co-IP, three adult C57BL/6J mice obtained from the Animal House Facility of the Universitat de Barcelona, as well as four wild type and four GABA_B1_ knockout mice (Schuler et al. 2001) from the Institute of Physiology, University of Basel, and three wild type, three GIRK2 knockout (Signorini et al. 1997) and three GIRK3 knockout (Torrecilla et al. 2002) mice from the University of Minnesota. Care and handling of animals prior to and during experimental procedures were in accordance with Japanese and European Union regulations (86/609/EC), and the protocols were approved by the local Animal Care and Use Committee.

### Antibodies and chemicals

The primary antibodies used were: rabbit anti-GABA_B1_ (B17, aa. 525–539 of mouse GABA_B1_; Kulik et al. 2002), guinea pig anti-Ca_V_2.1 (GP-Af810; aa. 361–400 of mouse Ca_V_2.1; Frontier Institute Co., Japan; Indriati et al. 2013), guinea pig anti-GIRK2 (GP-Af830; aa. 390–421 of mouse GIRK2; Frontier Institute Co., Japan; Aguado et al. 2008), rabbit anti-GIRK2 (Rb-Af280; aa. 390–421 of mouse GIRK2; Frontier Institute Co., Japan; Aguado et al. 2008), and rabbit anti-GIRK3 polyclonal (Rb-Af750; aa. 358–389 of mouse GIRK3; Frontier Institute Co., Japan; Aguado et al. 2008) polyclonal antibodies. ChromPure Rabbit IgG (011-000-003, Jackson ImmunoResearch Laboratories, Inc., West Grove, PA, USA) was used control IgG for coimmunoprecipitation experiments. The characteristics and specificity of the anti-GABA_B1_ antibody have been described elsewhere (Luján and Shigemoto 2006; Vigot et al. 2006). The characteristics and specificity of the anti-GIRK2 and anti-GIRK3 antibodies have been described elsewhere (Aguado et al. 2008; Fernández-Alacid et al. 2009). We have provided here further information on their specificity in the cerebellum using SDS-FRL. Indeed, to validate the specificity of the immunoreactions, GIRK2 knockout (KO) and GIRK3 KO mice were used. The pattern of immunoreactivity for GIRK2 and GIRK3 observed in the cerebellar cortex of wild-type mice was completely missing in that of the corresponding KO mice (see below). Secondary antibodies conjugated to 5 or 10 nm gold particles were purchased from British Biocell International (BBI, Cardiff, UK).

### Co-immunoprecipitation

A membrane suspension from the cerebella was obtained as described (Burgueño et al. 2003). In brief, membrane extracts were solubilised with radio-immunoprecipitation assay (RIPA) buffer (50 mM Tris–HCl (pH 7.4), 100 mM NaCl, 1% Triton-X 100, 0.5% sodium deoxycholate, 0.2% SDS and 1 mM EDTA) for 30 min on ice. The solubilised extract was then centrifuged at 13,000×*g* for 30 min and the supernatant (1 mg/mL) was processed for immunoprecipitation, each step of which was conducted with constant rotation at 0–4 °C. The supernatant was incubated overnight with the indicated antibody. Then 50 µL of TrueBlot™ anti-rabbit Ig IP Beads (eBioscience, San Diego, CA, USA) were added and the mixture was incubated overnight. Subsequently, the beads were washed with ice-cold RIPA buffer and aspirated to dryness with a 28-gauge needle. Then, 100 µL of sodium dodecyl sulphate-polyacrylamide gel electrophoresis (SDS-PAGE) sample buffer (0.125 M Tris–HCl pH 6.8, 4% SDS, 20% glycerol, and 0.004% bromophenol blue) was added to each sample. Immune complexes were dissociated by adding fresh dithiothreitol (DTT) (50 mM final concentration) and heating to 90 °C for 10 min. Proteins were resolved by SDS-PAGE on 7% polyacrylamide gels and then transferred to PVDF membranes using a semi-dry transfer system. The membranes were probed with the indicated primary antibody and a horseradish-peroxidase (HRP)-conjugated anti-guinea pig IgG or anti-rabbit IgG (Thermo Fisher Scientific, IL, USA). Immunoreactive bands were visualised using the chemiluminescence SuperSignal West Pico Chemiluminescent Substrate (Thermo Fisher Scientific Inc., Waltham, MA, USA) and detected in an Amersham Imager 600 (GE Healthcare Europe GmbH, Barcelona, Spain).

### SDS-digested freeze-fracture replica labelling (SDS-FRL) technique

SDS-FRL was performed with some modifications to the original method described previously (Fujimoto 1995). Animals were anesthetised with sodium pentobarbital (50 mg/kg, i.p.) and perfused transcardially with 25 mM PBS for 1 min, followed by perfusion with 2% paraformaldehyde in 0.1 M phosphate buffer (PB) for 12 min. The cerebella were dissected and cut into sagittal slices (130 µm) using a Microslicer (Dosaka, Kyoto, Japan) in 0.1 M PB. Next, we trimmed cerebellar slice middle lobules containing the molecular, PC and granule cell layers, and immersed them in graded glycerol of 10–30% in 0.1 M PB at 4 °C overnight. Slices were frozen using a high-pressure freezing machine (HPM010, BAL-TEC, Balzers). Slices were then fractured into two parts at − 120 °C and replicated by carbon deposition (5 nm thick), platinum (60° unidirectional from horizontal level, 2 nm), and carbon (15–20 nm) in a freeze-fracture replica machine (JFD II, JEOL). Replicas were transferred to 2.5% SDS and 20% sucrose in 15 mM Tris buffer (pH 8.3) for 18 h at 80 °C with shaking to dissolve tissue debris. The replicas were washed three times in 50 mM Tris-buffered saline (TBS, pH 7.4), containing 0.05% bovine serum albumin (BSA), and then blocked with 5% BSA in the washing buffer for 1 h at room temperature. Next, the replicas were washed and reacted with a polyclonal rabbit antibody for GABA_B1_ (5 μg/mL), a polyclonal guinea pig antibody for GIRK2 (8 μg/mL) and a rabbit antibody for GIRK3 (8 μg/mL), at 15 °C overnight. Following three washes in 0.05% BSA in TBS and blocking in 5% BSA/TBS, replicas were incubated in secondary antibodies conjugated with 10-nm gold particles overnight at room temperature. When the primary antibody was omitted, no immunoreactivity was observed. After immunogold labelling, the replicas were immediately rinsed three times with 0.05% BSA in BS, washed twice with distilled water, and picked up onto grids coated with pioloform (Agar Scientific, Stansted, Essex, UK). Co-localization of GABA_B1_ with effector ion channels was examined by double labelling with guinea pig antibodies against GIRK2 (Fernández-Alacid et al. 2009) and Ca_V_2.1 (Indriati et al. 2013). For double labelling of GABA_B1_ with GIRK2 or Ca_V_2.1, replicas were first reacted with the GABA_B1_ antibody (5 μg/mL) and then anti-rabbit secondary antibody, followed by incubation with the GIRK2 (8 μg/mL) or Ca_V_2.1 (8 μg/mL), antibodies and appropriate anti-guinea pig secondary antibody. After immunogold labelling, replicas were rinsed three times with 0.05% BSA/TBS, washed with TBS and distilled water, and picked up onto grids coated with pioloform (Agar Scientific).

### Development of automatic in-house software

**Fig. 1:**
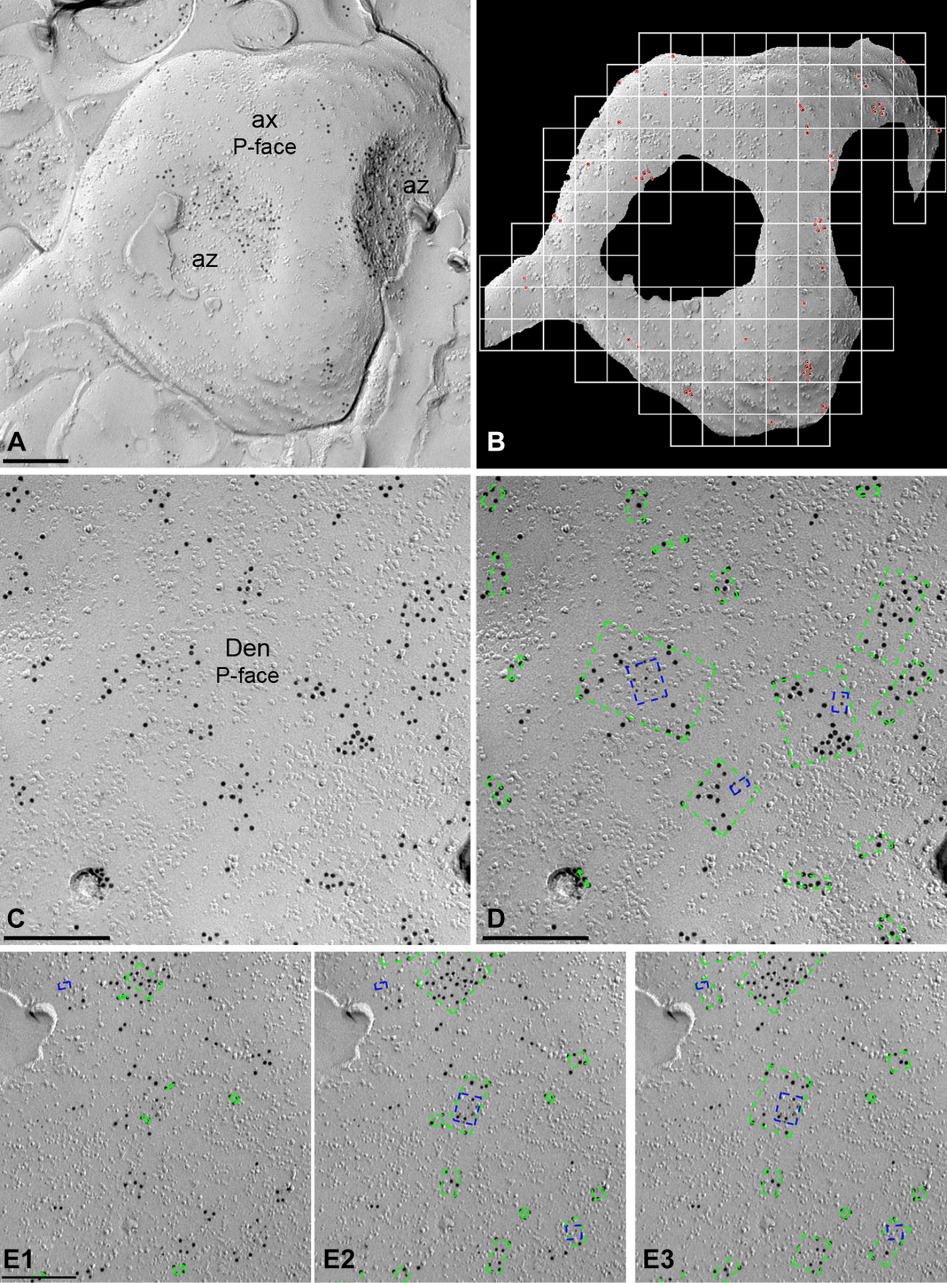
Development and operation of the GPDQ software used for quantitative analyses of immunoparticle distribution. **a** Image of an axon terminal (ax) with two active zones (az) in the molecular layer of the cerebellum immunolabelled for GABA_B1_ (10 nm) on the P-face. **b** To determine the density of immunoparticles, we first selected manually the contour of the compartment under study, and then the software calculates the area of the profile and the number of immunoparticles (red dots) per profile. **c** Image of a dendritic shaft (Den) of Purkinje cell double-labelled for GABA_B1_ (10 nm) and Ca_V_2.1 (5 nm) on the P-face in the molecular layer of the cerebellum. **d** The software determines the clustering according to the distance among gold particles, both for 10 nm (green dotted rectangles) and for 5 nm (blue dotted rectangles), establishing the number of immunoparticles and the distance among them. The dotted rectangles define the bounding box of the points on each cluster. **e1**–**e3** Clusters of immunoparticles were detected based on distance determined by standard deviation (SD) of NND. Mean (**e1**), mean + 1SD (**e2**) and mean + 2SD (**e3**) were tried and finally the mean + 2SD was chosen for data analysis. Scale bars: **a**–**e** 0.2 μm

We have developed *GPDQ* (*Gold Particle Detection and Quantification*), a software tool that performs automated and semi-automated detection of gold particles present in a given compartment of the cerebellum. The tool is interactive, allowing the user to supervise the process of segmentation and counting, modifying the appropriate parameters and validating the results as needed. It is also modular, which permits new functions to be implemented if required. We have also focused on usability, implementing a user-friendly interface to minimise the learning curve for the tool, and on portability, to make it accessible to a wide range of users (Fig. 1).

*GPDQ* has been implemented with MATLAB and Image Processing Toolbox 9.3 (The MathWorks, Inc., Natick, MA, USA). It is currently divided into four main modules: particle detection, analysis and simulation, graph and statistics generation, and visualisation. The particle detection module allows obtaining the radius and position (in nanometres from top-left corner) of the particles in the images. The automated version uses a two-stage procedure that detects the circles of a given diameter in the image with MATLAB’s implementation of the Hough transform (Yuen et al. 1990), and then determines which of them correspond to actual particles by means of a supervised classification model (Mitchel 1997). In particular, the default setting uses a Naïve Bayes classifier (Minsky 1961). Although the instantiation of the classifier (features and parameters) is provided in the software, it is possible to train and incorporate a different model. Due to the limitations of the Hough transform, which lacks for precision when particles are small, and the scales of the images, fully automated detection is only available for 10 nm particles at present. However, the tool provides a graphical interface for manual detection. It allows locating particles with any diameter even in rough surfaces. To make manual detection faster and more precise, the software automatically adjusts the position of each particle.

The second module allows for the processing of all information about images and particle locations, particle clusters and simulations. This module computes the number of particles, nearest neighbour distances (NNDs) to both particles of the same type (intra-type NNDs, e.g. from 5 nm particle to nearest 5 nm particle) and other type (inter-type NNDs, e.g. from 5 nm particle to nearest 10 nm particle). Clusters are obtained by single-linkage (Gower and Ross 1969). This method carries out an agglomerative hierarchical clustering, i.e. at first it considers each particle as a cluster, and iteratively merges the closest pair of clusters while the minimum *inter*-*cluster distance* (distance between a pair of clusters) is below a *threshold*. As inter-cluster distance measure, single-link considers the minimum distance between the pair of particles not yet belonging to the same cluster. As a consequence, any pair of particles at distance smaller than or equal to the minimum *inter*-*cluster distance threshold* belongs to the same cluster at the end of the process. The value of the threshold parameter was obtained from the distribution of the distances between each particle and its nearest neighbour. By default, the software uses mean + two times the standard deviation of such distances. Another important parameter is the *minimum number of particles in a cluster*, which was fixed to three. Thus, all clusters with one or two particles have been discarded. The software reports some information about the clusters, such as the number of particles, their area (as the area inside the convex hull of the particles in the cluster) or Ripley’s K function (Ripley 1976), or the distance to the nearest cluster of particles of either the same size (intra-cluster distance) or other size (inter-cluster distance). In addition, the second module allows for two types of simulations, termed random and fitted simulation: Random simulation removes all the particles of a given type from the image and redistributes them with two constraints: firstly, simulated particles cannot be within 10 nm of any other particle, and secondly, each pixel within the region of interest must have an equal probability of becoming the centre of a particle, while that probability is zero for each pixel outside of the region of interest. Fitted simulation, however, applies the additional constraint that the distribution of distances between simulated particles is not significantly different from the distribution of distances between the original particles. Similarity of distribution of distances is assessed by comparing all pairwise real and simulated distances by *Kolmogorov*–*Smirnov* (KS) test and considered similar if *p* ≥ 0.1. Otherwise, a particle is chosen at random and is randomly assigned to a new location within the area of interest and at least 10 nm away from other particles. Another KS test is performed and if the distances after this manipulation are less similar than before (indicated by a smaller *p* value), the particle is relocated to its previous location, and otherwise the new location is saved. This step is repeated until the constraint of a similar distance distribution between real and simulated particles (*p* ≥ 0.1) is satisfied. All measures consider the existence of two kinds of particles, separated according to their diameter. The software, however, is designed such that it can be quickly adapted for analysis of three or more kinds of particles. The third module deals with the generation of graphs and statistics, from the parameters computed with the second module. The fourth module allows for visualisation of the distribution of original particles as well as simulated particles as for example shown in Fig. 3c.

### Quantification and analysis of SDS-FRL data

The labelled replicas were examined using a transmission electron microscope (JEOL-1010) and photographed at magnifications of 60,000, 80,000, and 100,000. All antibodies used in this study were visualised by immunoparticles on the protoplasmic face (P-face), consistent with the intracellular location of their epitopes. Non-specific background labelling was measured on E-face surfaces.

#### Density gradient of GABA_B1_ along the neuronal surface

Quantitative analysis of immunogold labelling for GABA_B1_ was performed on three different dendritic compartments of PCs in the inner 1/3 of the ML, in PC somata in the PC layer and the axon terminals establishing synaptic contact with PC spines in the ML. The dendritic compartments analysed were the main apical dendrites, oblique dendrites and dendritic spines. Oblique dendrites were identified based on their small diameter and the presence of at least one emerging spine from the dendritic shaft. Dendritic spines were considered as such if: (1) they emerged from a dendritic shaft, or (2) they opposed an axon terminal recognised by the presence of synaptic vesicles on their cross-fractured portions. Axon terminals were identified based on: (1) the presence of synaptic vesicles in cross-fractures, or (2) the presence of an active zone, recognised by the concave shape of the P-face and the high density of IMPs. Non-specific background labelling was measured on E-face structures surrounding the measured P-faces. Images of the identified PC compartments were selected randomly over the entire dendritic tree of PCs and then captured with an ORIUS SC1000 CCD camera (Gatan, Munich, Germany). The area of the selected profiles and the number of immunoparticles were measured using our GPDQ software (Fig. 1a, b). Immunoparticle densities are presented as mean ± SD between animals. Statistical comparisons were performed with GraphPad Prism 5 software (La Jolla, CA, USA). Digitised images were then modified for brightness and contrast using Adobe PhotoShop CS5 (Mountain View, CA, USA) to optimise them for quantitative analysis.

#### Analysis of the spatial associations of GABA_B1_ receptors and GIRK2 or Ca_V_2.1 channels

For each of the molecules, we compared the mean intra-type NND of each image to the mean intra-type NNDs obtained from 500 random simulations generated from the same image. Individual images were considered significantly different from chance, if the real mean NND was within the lowest or highest 2.5% of the simulated mean NNDs, corresponding to a two-tailed test on a significance level of *α* = 0.05. They were considered associated when mean NND was within the lowest 2.5% and dissociated when mean NND was within highest 2.5% of the simulated mean NNDs. Lack of significant association or dissociation was concluded, when mean NND was within the remaining 95% of simulated mean NNDs. To assess whether a significant association exists for each compartment, we compared the real mean NNDs obtained from each image (*n* = 19–91) with 500 simulated mean NNDs by two-sided paired *t* test followed by Holm–Bonferroni correction for multiple testing, with a *p* < 0.05 being considered significant.

#### Analysis of colocalization between GABA_B1_ receptors and GIRK2 or Ca_V_2.1 channels

For each image, inter-type NNDs from 5 nm immunoparticles (GIRK2 or Ca_V_2.1) to10 nm gold particles (GABA_B1_) were measured using the GPDQ software. The mean NND was compared to 500 mean inter-type NNDs obtained from fitted simulations of 5 nm immunoparticles (GIRK2 or Ca_V_2.1) generated from the same image. Association or dissociation of 10 and 5 nm particles was considered significant in each image if the real mean NND was within the lowest or highest 2.5% of the simulated mean NNDs, corresponding to a two-tailed test on a significance level of *α* = 0.05. To assess whether a significant interaction exists as a whole for each compartment, we compared the real mean NNDs obtained from each image (*n* = 19–81) with 500 simulated mean NNDs by paired *t* test followed by Holm–Bonferroni correction for multiple testing, with a *p* < 0.05 being considered significant.

### Controls

To test method specificity in the procedures for SDS-FRL, antisera against GIRK2 and GIRK3 were tested on cerebellar slices of GIRK2 and GIRK3 knockout mice, respectively. In the replica samples, the immunogold signal disappeared completely in the knockout mouse cerebellum, while a strong signal was present in WT replicas. Furthermore, the primary antibody was either omitted or replaced with 5% (v/v) normal serum of the species of the primary antibody, resulting in total loss of the signal. To test for any cross-reactivity of secondary antibodies when double labelling was used by the SDS-FRL technique, some replicas were incubated with only one primary antibody and the full complement of the secondary antibodies. No cross-labelling was detected that would influence the results. In addition, some replicas were incubated with the two primary antibodies, but we swapped the size of immunogold in the secondary antibodies for the two targets proteins. No differences in distances of the two target proteins were detected that would influence our results. Finally, when double labelling was used, some replicas were incubated with a cocktail of two primary antibodies (GABA_B1_ and GIRK or Cav) followed by a cocktail of secondary antibodies. Other replicas were incubated with a primary antibody, and then incubated with the second primary antibody, followed by secondary antibodies, and other replicas were incubated with a changed sequence of primary antibodies, applying first primary antibody for GIRK or Cav followed by secondary antibody, and then we applied the second primary antibody (GABA_B1_) followed by secondary antibody. Under these conditions, we observed similar spatial distribution between two particles, hence that steric hindrance does not seem to affect interparticle distance.

### Data analysis

Statistical analyses for morphological data were performed using SigmaStat Pro (Jandel Scientific) and data were presented as mean ± SD) unless indicated otherwise. Statistical significance was defined as *p* < 0.05. The statistical evaluation of the immunogold densities was performed using the Kruskal–Wallis test, pairwise Mann–Whitney *U* test and Dunn’s method. Correlations were assessed using Pearson’s correlation test. To assess colocalisation between receptor and ion channels for each compartment, two-sided paired *t* test followed by Holm–Bonferroni correction for multiple testing was used.

## Results

### Immunoreactivity for GABA_B1_ is non-uniform in PCs

**Fig. 2:**
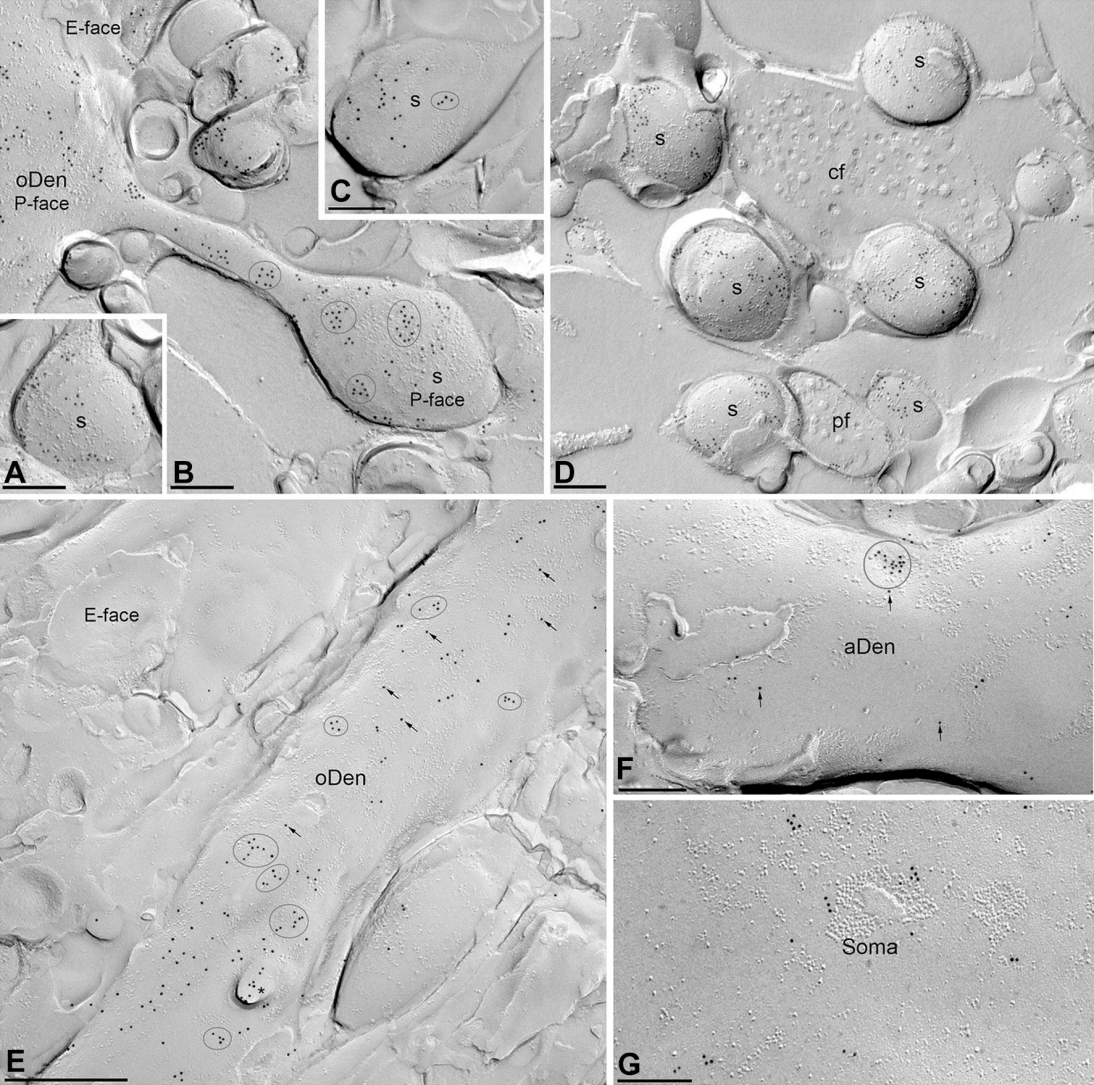
Somato-dendritic distribution of GABA_B1_ in PCs. Representative SDS-FRL electron micrographs of different compartments of PCs. **a**–**d** Clusters of GABA_B1_ immunoparticles (ellipses/circles) associated with the P-face were detected in dendritic spines (s) of PCs, both establishing synaptic contact with parallel fibres (pf) and climbing fibres (cf). Lower density of immunoparticles for GABA_B1_ was also detected scattered (arrows) outside those clusters. **e** In oblique dendrites (oDen), both clustered (ellipses/circles) and scattered (arrows) immunoparticles for GABA_B1_ were detected. Fractured spine necks are indicated with asterisks (*). The E-face is free of any immunolabelling. **f** In apical dendrites (aDen), we also detected clustered (circles) and scattered (arrows) immunoparticles for GABA_B1_, though at lower frequency. **g** A high-magnification image of a PC soma labelled for the GABA_B1_ subunit. Immunoparticles for GABA_B1_ in PC soma was low in density and always outside P-face IMP clusters. Scale bars: **a**–**d**, **f**, **g** 0.2 μm; **e** 0.5 μm

Using the pre-embedding immunogold method, we previously reported that GABA_B_ receptors are widely distributed in developing and adult PCs (Kulik et al. 2002; Luján and Shigemoto 2006). To accurately visualise the two-dimensional distribution of GABA_B_ receptors along somato-dendritic compartments of PCs, and to analyse receptor densities quantitatively, we used highly sensitive immunogold labelling in SDS-FRL (Masugi-Tokita and Shigemoto 2007) in this study. Electron microscopic analysis of the replicas revealed immunoparticles for the GABA_B1_ subunit on P-faces of PC plasma membranes (Fig. 2). Immunoparticles for GABA_B1_ were observed throughout the dendritic spines including the spine neck (Fig. 2a–d), dendritic shafts (Fig. 2e, f) and somata (Fig. 2g). The neuronal compartments that showed the highest density of immunoparticles for GABA_B1_ were dendritic spines, including those establishing synapses with parallel and climbing fibres (Fig. 2a–d). Regardless of the neuronal compartment, immunoparticles for GABA_B1_ were observed throughout the surface of PCs with two distinct patterns of distribution: scattered and clustered. The clustered pattern consists of aggregation of immunoparticles (> 3 gold particles) and the scattered pattern consists of isolated single immunoparticle detected on dendritic spines and shafts (Fig. 2a–f). Virtually no labelling was observed on the E-face (Fig. 2a–f) or on the cross-fracture of dendrites, spines or axon terminals.

**Fig. 3:**
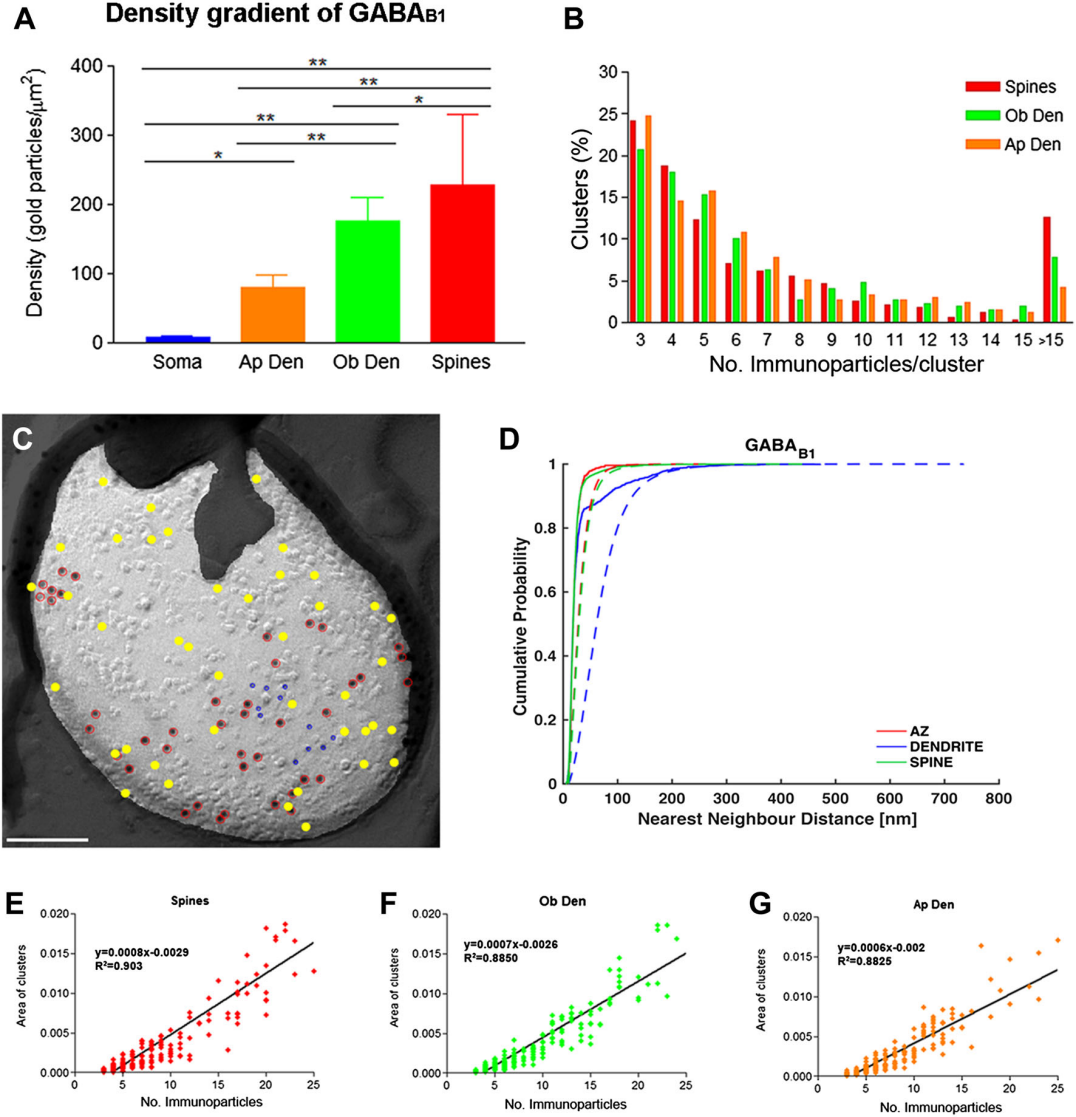
Density gradient and distribution profile of immunoparticles labelling GABA_B1_ along the surface of PCs. **a** Density of GABA_B1_ immunoparticles (including both isolated particles and those within small aggregations) increased from soma to distal dendrites (soma = 8.71 ± 1.43/μm^2^; apical dendrite = 79.14 ± 18.98/μm^2^; oblique dendrite = 175.33 ± 34.63/μm^2^; spines = 227.62 ± 102.18/μm^2^; Kruskal–Wallis test, pairwise Mann–Whitney *U* test and Dunn’s method, *p = 0.003; **p < 0.001). **b** The graph shows the quantification for the size of GABA_B1_ clusters in the spines, oblique dendrites and apical dendrites. Approximately 75% of clusters consisted of 3–8 immunoparticles. **c** Example showing random simulation of GABA_B1_ immunoparticles in a dendritic spine. Red: real GABA_B1_; Yellow: simulated GABA_B1_; blue: real GIRK2. Scale bar: 100 nm. **d** Cumulative probability plots of GABA_B1_ to GABA_B1_ NND. Solid and dotted lines show real and simulated GABA_B1_, respectively. *AZ* active zone. **e**–**g** Positive linear correlation was found between the number of GABA_B1_ immunoparticles and the area of clusters in the three dendritic compartments [spines, oblique dendrites (Ob Den) and apical dendrites (Ap Den)]

Next, we performed a quantitative comparison of the GABA_B1_ densities in different somato-dendritic compartments. A graded increase in the density of GABA_B1_ immunoparticles was found from the soma to the dendritic spines (Fig. 3a). Dendritic spines showed 26 times higher density of GABA_B1_ immunoparticles than soma, 3 times higher than apical dendrites and 1.2 times higher than oblique dendrites (Fig. 3a; *p* < 0.001 for soma vs. dendritic spines; *p* = 0.003 for dendritic spines vs. oblique dendrites; *p* < 0.001 for oblique dendrites vs. apical dendrites, Kruskal–Wallis test, pairwise Mann–Whitney *U* test, and Dunn’s method). We then conducted random simulations (Fig. 3c, d) to investigate whether GABA_B1_ immunoparticles were clustered. By comparing the NNDs between real and simulated particles from all images (Table 1), we found a highly significant clustering of GABA_B1_ immunoparticles in spines, dendrites and active zone (*p* < 0.001 for all compartments). We then asked whether we could detect significant clustering on individual images. We compared the mean NND of each image with the mean NNDs of the simulations, and judged the image to show a significant association if the real mean NND was within the smallest 2.5% of simulated mean NNDs or a significant dissociation if the real mean NND was within the largest 2.5% of simulated mean NNDs. We found that between 79 and 96% of profiles, depending on compartment, showed a significant association of GABA_B1_ immunoparticles with each other (Table 1), also indicating a clustered distribution of GABA_B1_. We further analysed the size and immunogold composition of clusters at different dendritic compartments. The size of clusters was similar between spines, oblique and apical dendrites, and quantification of immunoparticles revealed that around 75% of clusters were in the range of 3–8 immunoparticles (Fig. 3b). In these three compartments, the surface area of clusters (defined by the software) and the immunoparticle number showed a strong positive linear correlation (Fig. 3e–g; *r* = 0.864, 0.884, 0.866 for spines, oblique dendrites, and apical dendrites, respectively), indicating constant density of GABA_B1_ across clusters.

**Table 1:** Clustered distribution of GABA_B1_, GIRK2 and Ca_V_2.1 in active zones and dendritic shafts and spines

Molecule	Compartment	Real NND	Simulated NND	*p* value	Association (%)	*N*
GABA_B1_	Active zone	22.5 ± 5.6	35.8 ± 13.2	2.3E−04	78.9	19
	Dendrite	38.9 ± 17.4	83.2 ± 28.6	2.8E−10	96.0	25
	Spine	27.1 ± 16.5	44.9 ± 26.2	5.3E−19	89.0	91
GIRK2	Active zone	19.8 ± 5.2	48.7 ± 23.3	8.7E−05	89.5	19
	Dendrite	43.2 ± 29.2	204.8 ± 123.9	5.3E−06	100.0	24
	Spine	30.6 ± 22.2	80.6 ± 37.7	1.2E−24	83.0	88
Ca_V_2.1	Active zone	20.0 ± 5.8	37.3 ± 24.4	3.9E−04	97.1	35
	Dendrite	36.9 ± 16.9	141.8 ± 102.6	2.4E−04	100.0	26
	Spine	27.6 ± 17.5	66.0 ± 33.5	1.5E−04	84.2	19

NNDs are reported as mean ± standard deviation of image means, in case of simulations, image means are the means over all 500 simulations of that image. *p* values were obtained by two-sided paired *t* test followed by Holm–Bonferroni correction. “Association” shows the percentage of image means within the lowest 2.5% of simulation means. In none of the images did we detect a significant dissociation, i.e. a mean NND within the highest 2.5% of simulated mean NNDs. *N* indicates the number of images used for analysis

### Coupling of GABA_B1_ receptors with GIRK and Ca_V_2.1 channels in cerebellar membranes

**Fig. 4:**
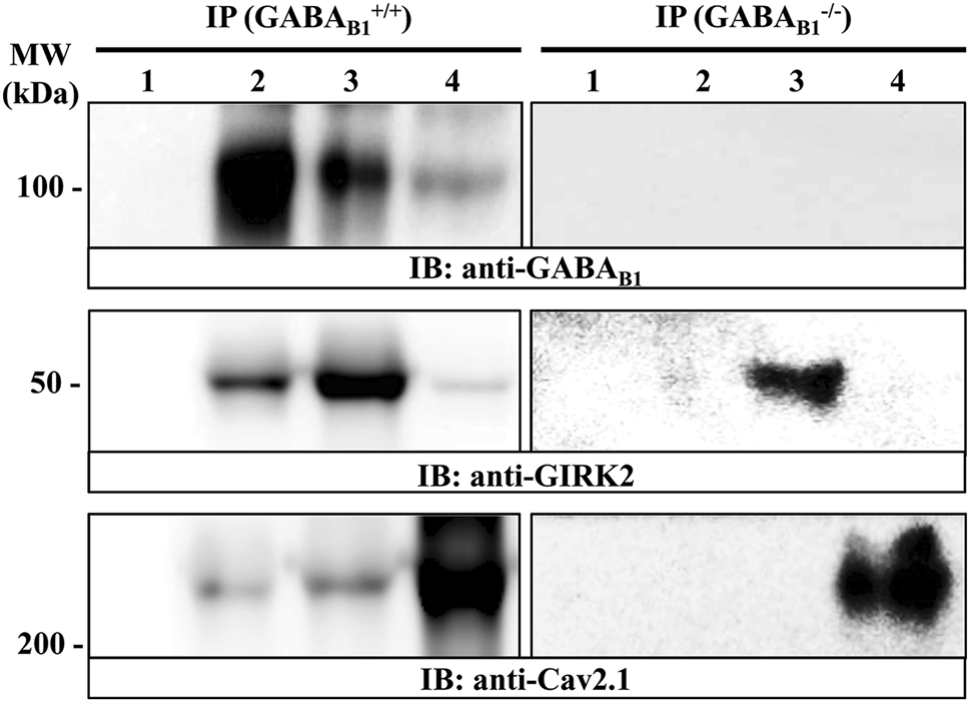
Co-immunoprecipitation of GABA_B1_ receptor and GIRK2 and Ca_V_2.1 channels from mouse cerebellum. Solubilised cerebellar membrane extracts from wild type (+/+) and GABA_B1_ receptor knock-out (−/−) mice were subjected to immunoprecipitation analysis using control rabbit IgG (2 μg, lane 1), rabbit anti-GABA_B1_ (2 μg, lane 2), rabbit anti-GIRK2 (2 μg, lane 3) and rabbit anti-Ca_V_2.1 (2 μg, lane 4). Immunoprecipitates (IP) were analysed by SDS-PAGE and immunoblotted using a rabbit anti-GABA_B1_ (1 μg/mL), guinea pig anti-GIRK2 (1 μg/mL) and guinea pig anti-Ca_V_2.1 (1 μg/mL). Immunoreactive bands were detected as described in experimental procedures

To assess the formation of putative macromolecular complexes containing GABA_B1_ receptor and its effector molecules, namely GIRK channel and Ca_V_2.1 channel, co-immunoprecipitation experiments were performed. Accordingly, using soluble membrane extracts from mouse cerebellum the anti-GABA_B1_, the anti-GIRK2 and the anti-Ca_V_2.1 antibodies were able to immunoprecipitate a band of ~ 100 kDa (Fig. 4, lane 2, IP: GABA_B1_^+/+^, IB: anti-GABA_B1_), ~ 50 kDa (Fig. 4, lane 3, IP: GABA_B1_^+/+^, IB: anti-GIRK2) and ~ 250 kDa (Fig. 4, lane 4, IP: GABA_B1_^+/+^, IB: anti-Ca_V_2.1) which correspond to GABA_B1_, GIRK2 and Ca_V_2.1 subunits, respectively. Interestingly, the anti-GABA_B1_ antibody was able to co-immunoprecipitate the GIRK2 channel (Fig. 4: lane 2, IP: GABA_B1_^+/+^, IB: anti-GIRK2), as expected (Ciruela et al. 2010), and the Ca_V_2.1 channel (Fig. 4: lane 2, IP: GABA_B1_^+/+^, IB: anti-Ca_V_2.1). On the other hand, the anti-GIRK2 antibody co-immunoprecipitated the GABA_B1_ receptor and the Ca_V_2.1 channel (Fig. 4, lane 3, IP: GABA_B1_^+/+^, IB: anti-GABA_B1_ and IB: anti-Ca_V_2.1, respectively), and the anti-Ca_V_2.1 antibody co-immunoprecipitated the GABA_B1_ receptor and the GIRK2 channel (Fig. 4, lane 4, IP: GABA_B1_^+/+^, IB: anti-GABA_B1_ and IB: anti-GIRK2, respectively). Importantly, these bands did not appear when an irrelevant rabbit IgG (control IgG) was used for immunoprecipitation (Fig. 4, lane 1), showing that the immunoprecipitation was specific. In addition, when the co-immunoprecipitation experiments were performed using soluble extracts from GABA_B1_ receptor knockout mouse cerebellum the anti-GABA_B1_ antibody, unable to immnoprecipitate the GABA_B1_ receptor (Fig. 4: lane 2, IP: GABA_B1_^−/−^, IB: anti-GABA_B1_), did not co-immunoprecipitate neither the GIRK2 channel nor the Ca_V_2.1 channel (Fig. 4: lane 2, IP: GABA_B1_^−/−^, respectively; IB: anti-GIRK2 and IB: anti-Ca_V_2.1, respectively).

In addition, in the very same soluble extract, the anti-GIRK2 and the anti-Ca_V_2.1 antibodies only immunoprecipitated the GIRK2 channel and the Ca_V_2.1 channel, respectively (Fig. 4, lane 3, IP: GABA_B1_^−/−^, IB: anti-GIRK2 and lane 4, IP: GABA_B1_^−/−^, IB: anti-Ca_V_2.1, respectively). Of note, the absence of orthogonal GIRK2 and Ca_V_2.1 co-immunoprecipitation from GABA_B1_^−/−^ cerebellar extracts might reveal an essential role of GABA_B_ receptor nucleating the GIRK2/GABA_B_/Ca_V_2.1 heterocomplex, yet this contention will need to be further studied in the future. Alternatively, a lack of sensitivity of either the immunoprecipitation and/or immunoblot process would also explain the absence of orthogonal GIRK2 and Ca_V_2.1 co-immunoprecipitation. Overall, these results suggest that in mouse cerebellum, GABA_B_ receptor/GIRK channel, GABA_B_ receptor/Ca_V_2.1 channel might assemble into stable protein–protein complexes resistant to co-immunoprecipitation processing, thus reinforcing the idea that these oligomeric complexes might have physiological relevance in vivo.

### Preferential localization of GABA_B1_ with GIRK channels in PC spines

**Fig. 5:**
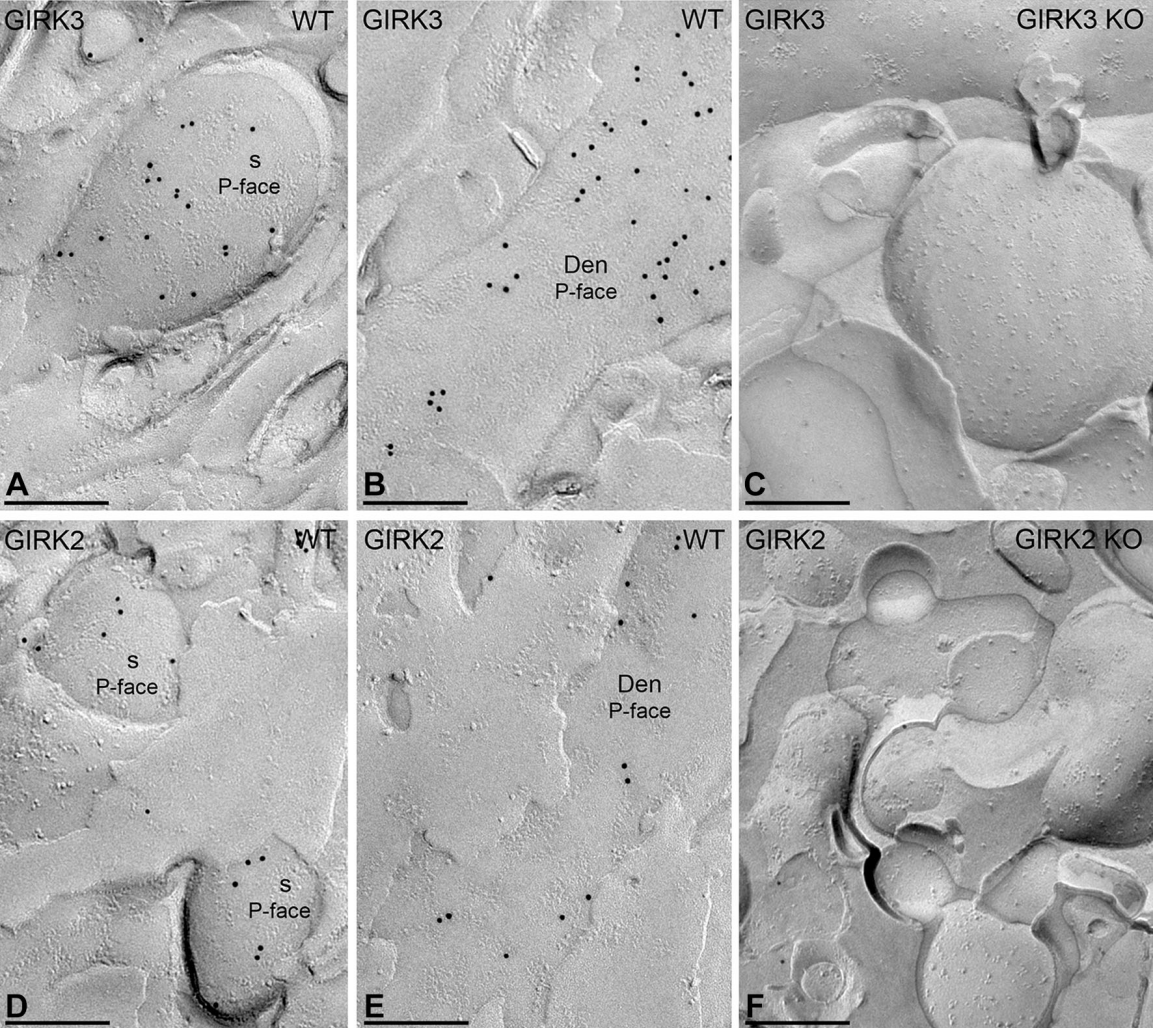
Subcellular localization for GIRK channel subunits in PCs. Distribution of immunoparticles for the GIRK3 and GIRK2 subunits using the SDS-FRL technique in wild-type (WT) and GIRK knockout (KO) mice. **a**, **b** Immunoparticles for the GIRK3 subunit are detected in dendritic spines (s) and dendritic shafts of oblique dendrites (oDen) of PCs. **c** The antibody specificity was controlled and confirmed in replicas of GIRK3 KO mice that were free of any immunolabelling. **d**, **e** Immunoparticles for the GIRK2 subunit are also detected in dendritic spines (s) and dendritic shafts (Den) of PCs, but at lower frequency than GIRK3. **f** The immunogold labelling for GIRK2 was abolished in the GIRK2 KO mice. Scale bars: **a**–**f** 0.2 μm

We previously reported the molecular interaction between GABA_B_ receptors and GIRK channels in the cerebellum (Fernández-Alacid et al. 2009; Ciruela et al. 2010). PCs express GIRK1, GIRK2 and GIRK3, although the most predominant subunit is GIRK3 (Aguado et al. 2008; Fernández-Alacid et al. 2009). To provide morphological insights into the GABA_B_–GIRK interaction, we carried out double-labelling SDS-FRL experiments. Since our anti-GIRK3 antibody was raised in the same species as our anti-GABA_B1_ antibody, we performed double-labelling SDS-FRL experiments with an anti-GIRK2 antibody only. However, we first compared the subcellular distribution of GIRK2 and GIRK3 in PCs. In single-labelling experiments, immunoparticles for GIRK3 (Fig. 5a, b) and GIRK2 (Fig. 5d, e) were found on the P-face of dendritic shafts and spines. These immunogold labelling patterns were abolished in the GIRK3 KO (Fig. 5c) and GIRK2 KO (Fig. 5f) mice, respectively. Thus, we conclude that GIRK2 and GIRK3 exhibit comparable subcellular distributions in PCs.

**Fig. 6:**
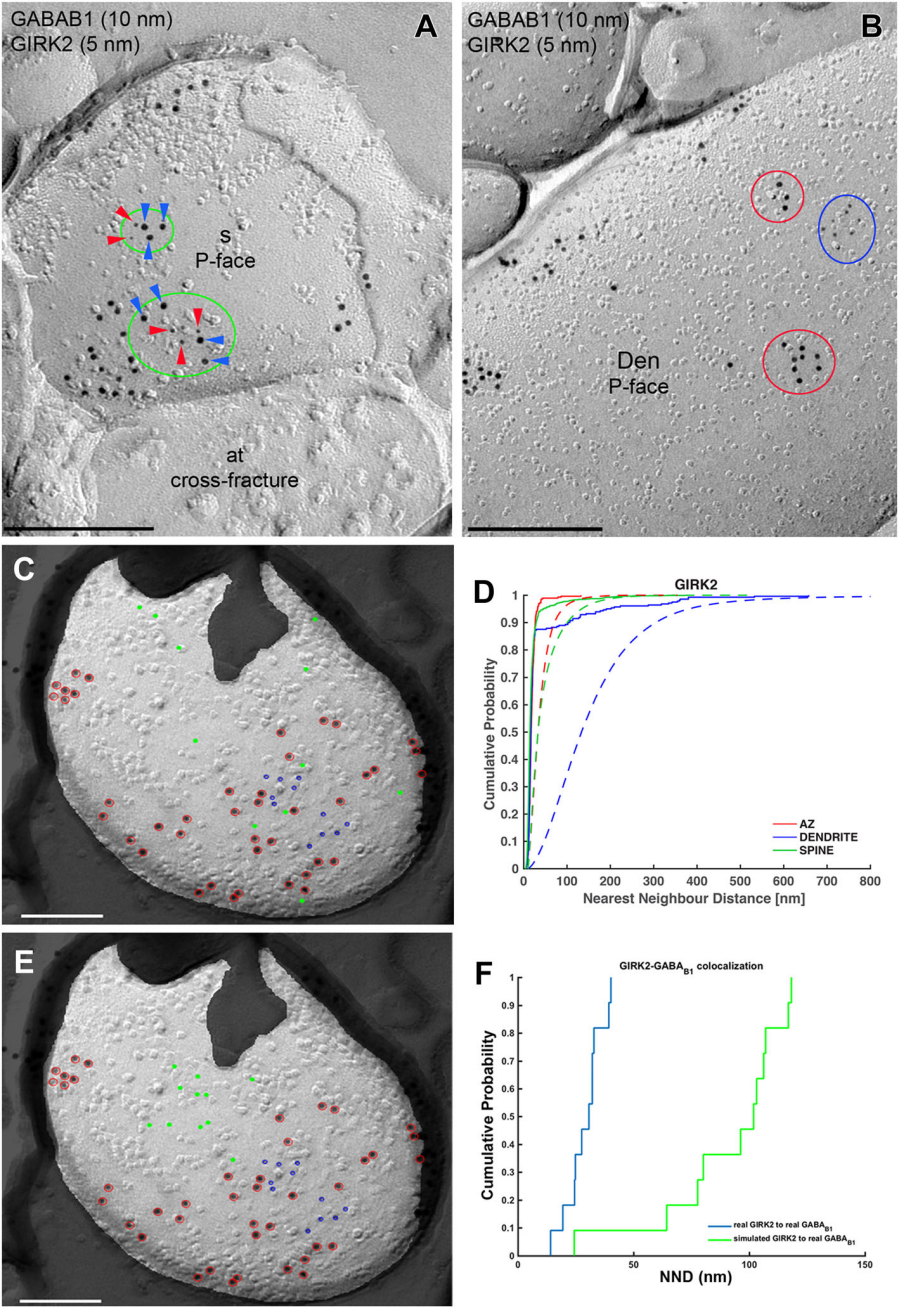
Compartment-dependent co-localization of GABA_B1_ with GIRK2. Electron micrographs showing double-labelling for GABA_B1_ (10 nm) and GIRK2 (5 nm) in PCs, as detected using the SDS-FRL technique. **a** In dendritic spines (s), immunoparticles for GIRK2 (red arrowheads) co-clustered (green ellipses) with those for GABA_B1_ (blue arrowheads). **b** In dendritic shafts (Den) of PCs, clusters of GABA_B1_ immunoparticles (red ellipses) were segregated from clusters of GIRK2 immunoparticles (blue ellipses). Red, green and blue ellipses were drawn manually using Adobe Photoshop for illustration purposes, to show the particles corresponding to clusters. They do not represent the area of clusters as defined using *GPDQ* software and not all of the clusters detected are shown. **c** Example showing random simulation of GIRK2 immunoparticles in a dendritic spine. Blue: real GIRK2; red: real GABA_B1_; green: simulated GIRK2. **d** Cumulative probability plots of GIRK2 to GIRK2 nearest neighbour distance (NND). Solid and dotted lines show real and simulated GIRK2, respectively. **e** Example showing fitted simulation of GIRK2 immunoparticles in a dendritic spine. Blue: real GIRK2; red: real GABA_B1_; green: simulated GIRK2. **f** Cumulative probability plot showing GIRK2 to GABA_B1_ NND of the particular simulation shown in **e**. *AZ* active zone. Scale bars: **a**, **b** 200 nm; **c**, **e** 100 nm

Next, we performed double-labelling experiments for GABA_B1_ and GIRK2 (Fig. 6). Immunoparticles for GABA_B1_ appeared co-clustered with those for GIRK2 along the extrasynaptic plasma membrane of dendritic spines (Fig. 6a), but not on dendritic shafts, where clusters of GABA_B1_ immunoparticles seemed mostly segregated from those of GIRK2 (Fig. 6b). To quantitatively analyse the extent of the spatial relationship between GABA_B1_ and GIRK2, we first asked whether GIRK2 immunoparticles themselves showed a clustered distribution. We compared NNDs of random simulations of GIRK2 immunoparticles with the real distributions (Fig. 6c, d) and found significant clustering in all compartments (*p* < 0.001 for all compartments), with 83–100%, depending on compartment, of individual profiles showing a significant association (Table 1). To understand the spatial relationship between GABA_B1_ and GIRK2, we then conducted fitted simulations (see “Materials and methods”) of GIRK2 immunoparticles (Fig. 6e) to keep the spatial relationship among GIRK2 immunoparticles as close to reality as possible. We then compared NNDs from real and simulated GIRK2 particles to real GABA_B1_ particles in dendritic shafts and spines (Fig. 6f), and found a significant association between GABA_B1_ and GIRK2 in dendritic spines (*p* < 0.001), while we observed a significant dissociation in dendritic shafts (*p* < 0.01) (Table 2). On the level of individual dendritic spines, we found that 21% showed a significant association, while 1% showed a significant dissociation. For dendritic shafts, we found a significant dissociation in 50% of profiles and a significant association in 4% of profiles. Consistent with these results, we found that inter-cluster distances between GIRK2 and GABA_B1_ clusters were significantly smaller in dendritic spines compared with dendritic shafts (Table 3).

**Table 2:** Spatial relationship of GABA_B1_ with GIRK2 and Ca_V_2.1

Simulated molecule	Compartment	Real NND	Simulated NND	*p* value	Association (%)	Dissociation (%)	*N*
GIRK2	Active zone	42.4 ± 13.8	47.0 ± 18.4	0.36	5.3	5.3	19
	Dendrite	158.4 ± 78.4	119.2 ± 48.8	0.0088	4.2	50.0	24
	Spine	42.4 ± 20.9	59.2 ± 22.5	4.1E−14	21.3	1.1	89
Ca_V_2.1	Active zone	41.8 ± 11.6	43.1 ± 11.4	0.77	14.3	2.9	35
	Dendrite	44.0 ± 17.8	62.9 ± 13.7	3.4E−08	46.2	0.0	26
	Spine	98.0 ± 37.7	96.5 ± 34.5	0.83	10.0	10.0	20

NNDs are reported as mean ± standard deviation of image means, in case of simulations, image means are the means over all 500 simulations of that image. *p* values were obtained by two-sided paired *t* test followed by Holm–Bonferroni correction. “Percent significant” show the percentage of images whose mean NNDs are within the top or bottom 2.5% of simulation means. “Dissociation” and “Association” show the percentage of image means within the top and bottom 2.5%, respectively, of simulation means. *N* indicates the number of images used for analysis

**Table 3:** Spatial relationship between Ca_V_2.1 or GIRK2 and GABA_B1_ clusters

Clusters	Compartment	Inter-cluster distance	*N*
GIRK2 to GABA_B1_	Active zone	84.2 ± 46.1	21
	Dendrite	219.6 ± 119.0	54
	Spine	110.7 ± 73.4	100
Ca_V_2.1 to GABA_B1_	Active zone	68.5 ± 45.1	44
	Dendrite	124.2 ± 126.7	85
	Spine	154.9 ± 85.9	31

Inter-cluster distances from Ca_V_2.1 or GIRK2 clusters to the nearest neighbouring GABA_B1_ clusters are reported as mean ± standard deviation. Inter-cluster distances between GIRK2 and GABA_B1_ in dendritic shafts differed significantly from both the ones in spines (*p* < 0.001) and in active zones (*p* < 0.001), while there was no significant difference between inter-cluster distances in spines and active zones (*p* = 0.12). Inter-cluster distances between Ca_V_2.1 and GABA_B1_ were showed significant differences between active zones and dendritic shafts (*p* = 0.004), active zones and dendritic spines (*p* < 0.001) as well as dendritic shafts and spines (*p* = 0.009). *p* values were obtained by Mann–Whitney *U* test followed by Holm–Bonferroni correction. *N* indicates the number of Ca_V_2.1 or GIRK clusters analysed

### Preferential localization of GABA_B1_ with Ca_V_2.1 channels in PC dendrites

**Fig. 7:**
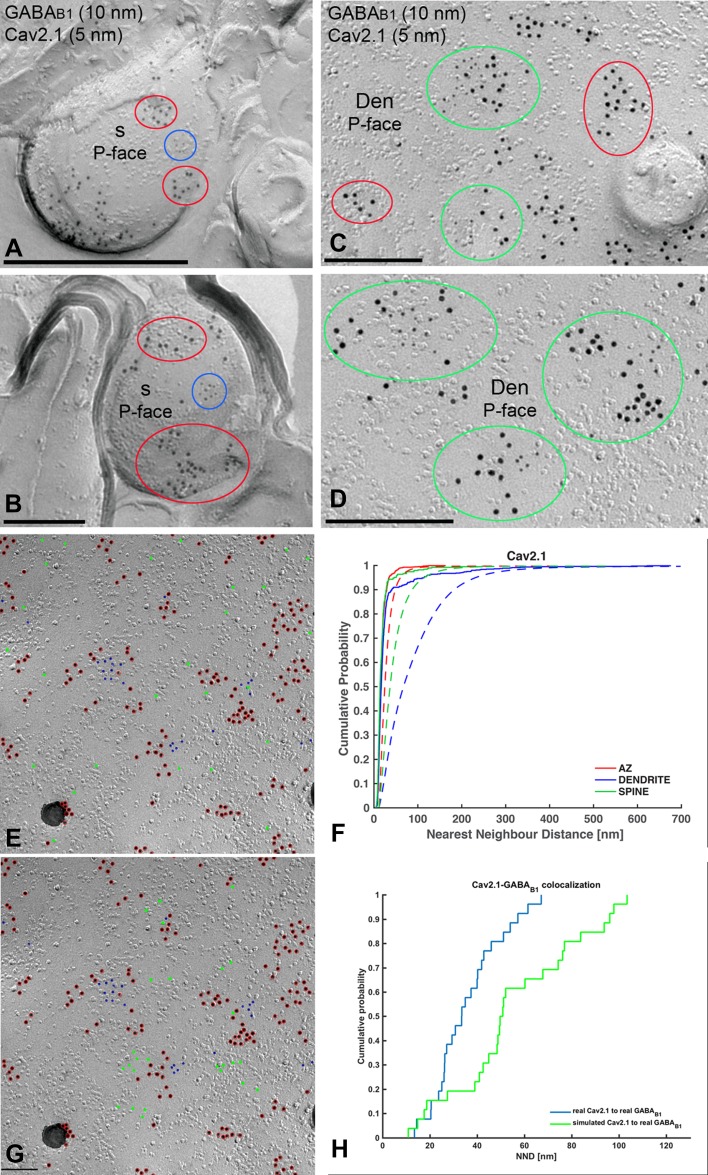
Preferential colocalisation of GABA_B1_ with Ca_V_2.1 in PC dendritic shafts. Electron micrographs showing double-labelling for GABA_B1_ (10 nm) and Ca_V_2.1 (5 nm) in PCs, as detected using the SDS-FRL technique. **a**, **b** In dendritic spines (s), clusters of GABA_B1_ immunoparticles (red ellipses) are close to but mostly segregated from clusters of Ca_V_2.1 immunoparticles (blue ellipses). **c**, **d** In dendritic shafts (Den), immunoparticles for GABA_B1_ immunoparticles co-clustered with immunoparticles for Ca_V_2.1 (green ellipses). Clusters of GABA_B1_ immunoparticles without immunoparticles for Ca_V_2.1 (red ellipses) were also found in dendritic spines of PCs. Red, green and blue ellipses were drawn manually using Adobe Photoshop for illustration purposes, to show the particles corresponding to clusters. They do not represent the area of clusters as defined using *GPDQ* software and not all of the clusters detected are shown. **e** Example showing random simulation of Ca_V_2.1 immunoparticles in a dendritic shaft. Blue: real Ca_V_2.1; red: real GABA_B1_; green: simulated Ca_V_2.1. **f** Cumulative probability plots of Ca_V_2.1 to Ca_V_2.1 NND. Solid and dotted lines show real and simulated Ca_V_2.1, respectively. **g** Example showing fitted simulation of Ca_V_2.1 immunoparticles in a dendritic shaft. Blue: real Ca_V_2.1; red: real GABA_B1_; green: simulated Ca_V_2.1. **h** Cumulative probability plot showing Ca_V_2.1 to GABA_B1_ NND of the particular simulation shown in **g**. *AZ* active zone. Scale bars: **a**–**d** 200 nm; **e**, **g** 100 nm

We next performed double-labelling experiments for GABA_B1_ and Ca_V_2.1 (Fig. 7). Immunoparticles for Ca_V_2.1 were found on the P-face of dendritic shafts and spines, but not on the E-face or on cross-fractures (Fig. 7a–d). Immunoparticles for GABA_B1_ were close to those for Ca_V_2.1 but seemed not co-clustered along the extrasynaptic plasma membrane of dendritic spines (Fig. 7a, b). In contrast, in dendritic shafts GABA_B1_ immunoparticles appeared co-clustered with those for Ca_V_2.1 (Fig. 7c, d), although many clusters of GABA_B1_ immunoparticles were not apparently associated with clusters of Ca_V_2.1 immunoparticles (Fig. 7c). Random simulations of Ca_V_2.1 (Fig. 7e, f) showed a significant clustering of Ca_V_2.1 immunoparticles with themselves in all compartments (*p* < 0.001), with 84–100%, depending on compartment, of individual profiles showing significant association (Table 1). To quantify their extent of spatial relation, the NNDs between immunoparticles for GABA_B1_ and Ca_V_2.1 were compared with those between real GABA_B1_ and simulated Ca_V_2.1 particles (fitted simulations, see “Materials and methods”) (Fig. 7g, h). We found a significant association of GABA_B1_ with Ca_V_2.1 in dendritic shafts (*p* < 0.001). In dendritic spines, however, co-clustering occurred only on chance level (*p* = 0.83) (Table 2). On the level of individual dendritic spines, we found that 10% each showed significant association or dissociation. For dendritic shafts, we found a significant association in 46% of profiles and no profile showing significant dissociation. In line with these results, we found that inter-cluster distances between Ca_V_2.1 and GABA_B1_ clusters were significantly smaller in dendritic shafts compared with dendritic spines (Table 3).

### GABA_B1_ is not co-clustered with but close to GIRK or Ca_V_2.1 channels at presynaptic sites

**Fig. 8:**
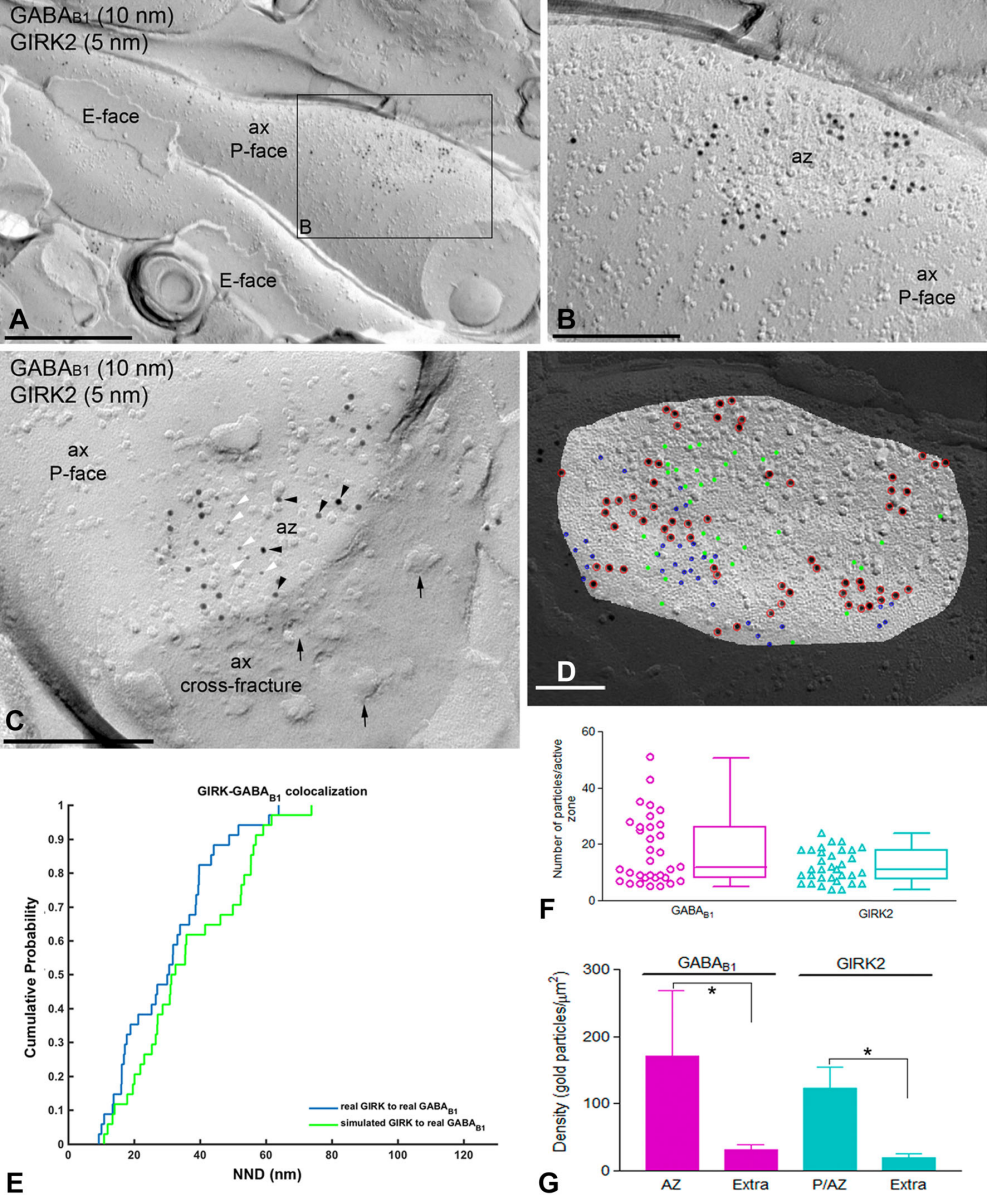
Co-distribution of GABA_B1_ and GIRK2 within the presynaptic active zone of axon terminals. Electron micrographs showing the P-face and cross-fracture of axon terminals (ax) identified by presence of synaptic vesicles (arrows) and active zones (az), recognised by the concave shape of the P-face and the high density of IMPs. **a** A low-magnification image of an axon terminal (ax) with synaptic contact co-labelled for GABA_B1_ and the GIRK2. **b** Higher magnification image of the boxed area shown in **a**. Small clusters of immunogold particles for GABA_B1_ (10 nm) were mostly found at the edge of active zones co-distributed with immunogold particles for GIRK2 (5 nm). **c** In a few axon terminals, immunoparticles for GABA_B1_ (black arrowheads) co-distributed with immunoparticles for GIRK2 (white arrowheads) in the active zone (az). **d** Example showing fitted simulation of GIRK2 immunoparticles in an active zone. Blue: real GIRK2; red: real GABA_B1_; green: simulated GIRK2. **e** Cumulative probability plot showing GIRK2 to GABA_B1_ NNDs of the particular simulation shown in **d**. **f** High variability of number of GABA_B1_ immunoparticles (range 5–51) and GIRK2 immunoparticles (range 4–24) was found at the edge and inside of active zones. Box chart shows fifth, 25th, 75th, and 95th percentiles and median (bar). **g** Densities of GABA_B1_ and GIRK2 immunoparticles at the active zone and extrasynaptic areas of axons. The density of both GABA_B1_ and GIRK2 immunoparticles was significantly higher at the edge and inside of active zones (P/AZ; GABA_B1_ = 171.66 ± 97.03/μm^2^; GIRK2 = 123.36 ± 31.91/μm^2^) than at extrasynaptic sites (Extra; GABA_B1_ = 31.06 ± 8.26/μm^2^; GIRK2 = 19.83 ± 5.90/μm^2^) (Kruskal–Wallis test, pairwise Mann–Whitney *U* test and Dunn’s method, *p < 0.001). Scale bars: **a** 0.5 μm; **b**, **c** 0.2 μm; **d** 0.1 µm

Immunoparticles for GABA_B1_ were not only confined to somato-dendritic domains of PCs, but also present in axon terminals, as previously reported (Kulik et al. 2002; Luján and Shigemoto 2006). Accordingly, we analysed whether GABA_B_ receptors co-clustered with their effector ion channels at presynaptic sites. First, we examined the co-clustering and spatial relationship of GABA_B1_ with GIRK2 channels. Most immunoparticles for GIRK2 were detected close to active zones (Fig. 8a–c), and in some cases concentrated in the active zone of axon terminals (Fig. 8c). The NNDs from GIRK2 to GABA_B1_ immunoparticles in the active zone were as short as that (42.4 nm) found in dendritic spines (Table 1). However, we found no difference (*p* = 0.36) between real NND and that from simulated GIRK2 to GABA_B1_ immunoparticles (Fig. 8d, e), indicating absence of significant co-clustering. In active zones, 5% of individual profiles showed association and 5% showed dissociation (Table 2). The inter-cluster distance between GIRK2 and GABA_B1_ clusters, however, was not significantly different between active zones and dendritic spines, where a significant association had been detected (Table 3). In addition, the number of immunoparticles was highly variable and ranged from 5 to 51 per active zone for GABA_B1_ (Fig. 8f; mean = 17.8, median = 12, interquartile range = 8–26.25, *n* = 33 active zone profiles from three animals), and ranged from 4 to 24 per active zone for GIRK2 (Fig. 8f; mean = 12.2, median = 11, interquartile range = 7.75–18, *n* = 33 active zone profiles from three animals). The density of GABA_B1_ and GIRK2 at the active zone was 171.66 ± 97.02 and 123.36 ± 31.91 immunogold/μm^2^, respectively (Fig. 8g). Low densities of GABA_B1_ (mean = 31.1 immunogold/μm^2^, median = 34, interquartile range = 26.1–35.9, *n* = 15 profiles from three animals) and GIRK2 (mean = 19.8 immunogold/μm^2^, median = 22.3, interquartile range = 18.2–23.7, *n* = 15 profiles from three animals) were in axon terminals (Fig. 8g), indicating accumulation of both GABA_B_ receptors and GIRK2 in the active zone.

**Fig. 9:**
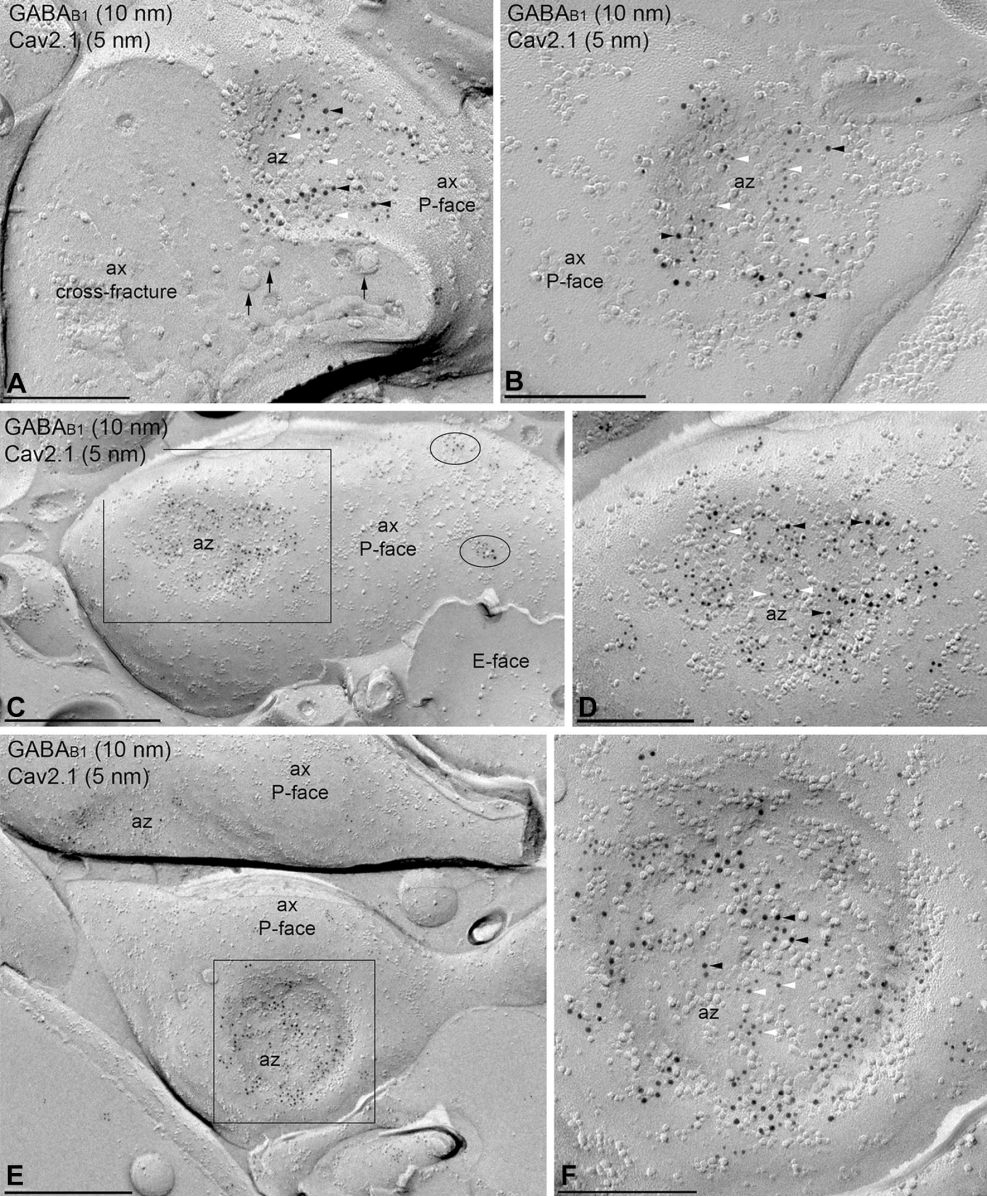
Co-distribution of GABA_B1_ and Ca_V_2.1 in the presynaptic active zone of axon terminals. **a**–**f** Electron micrographs showing the P-face and cross-fracture of axon terminals (ax) identified by presence of synaptic vesicles (arrows) and active zones (az) recognised by the concave shape of the P-face and the high density of IMPs. Black boxes in **c** and **e** represent images enlarged in **d** and **f**, respectively. Immunoparticles for GABA_B1_ (10 nm, black arrowhead) were found within the active zone (az) co-distributed, but not co-clustered, with immunoparticles for Ca_V_2.1 (5 nm, white arrowhead). In a few axon terminals (**b**), immunoparticles for GABA_B1_ co-distributed with immunoparticles for Ca_V_2.1 only at the edge but not in the central part of the active zone. Few clusters of GABA_B1_ and Ca_V_2.1 were detected at extrasynaptic sites of axon terminals (black ellipses in **c**). Scale bars: **a**, **b**, **d**, **f** 0.2 μm; **c**, **e** 0.5 μm

**Fig. 10:**
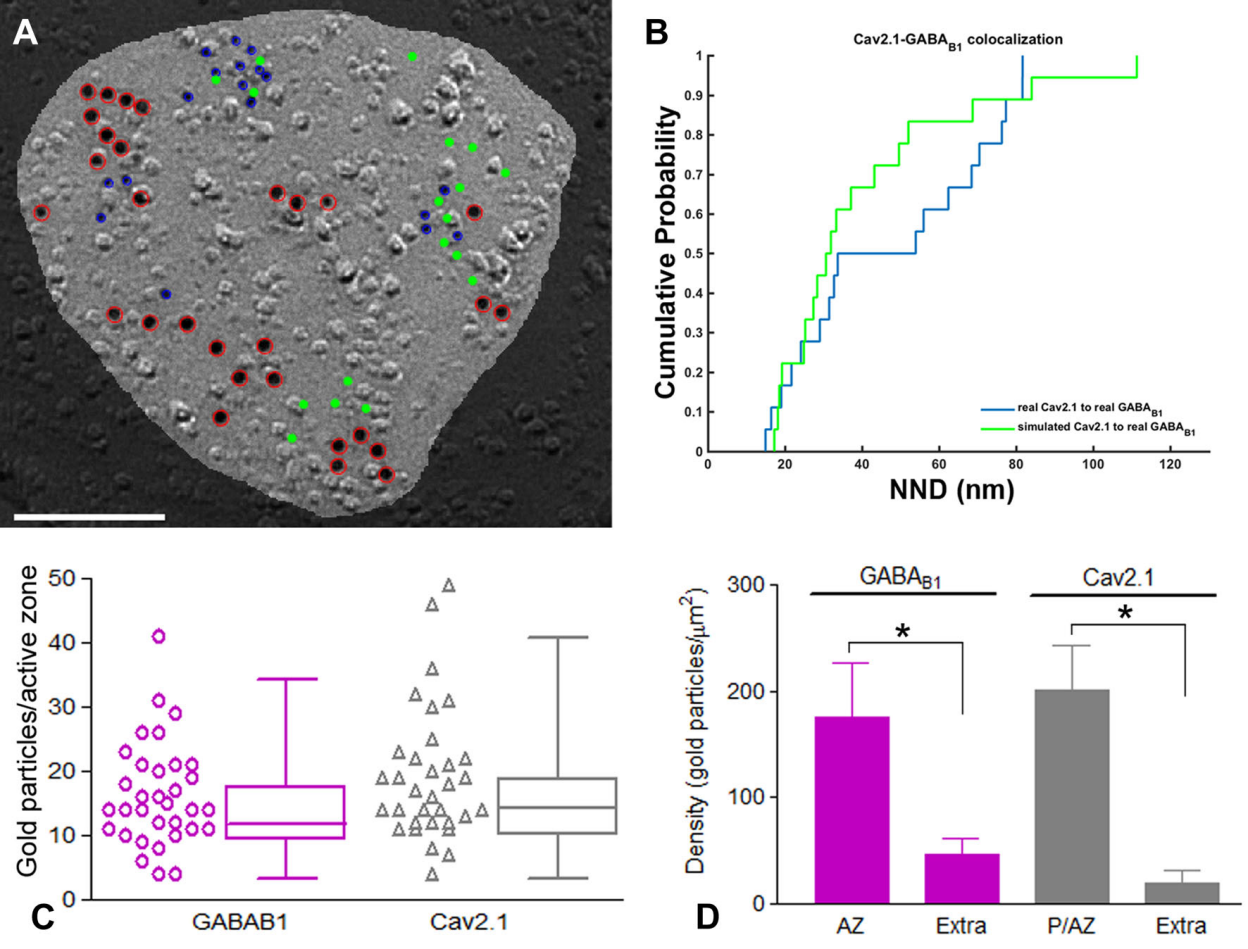
Variability and density of GABA_B1_ and Ca_V_2.1 in active zone and axon terminals. **a** Example showing fitted simulation of Ca_V_2.1 immunoparticles in an active zone. Blue: real Ca_V_2.1; red: real GABA_B1_; green: simulated Ca_V_2.1. Scale bar, 100 nm. **b** Cumulative probability plot showing Ca_V_2.1 to GABA_B1_ NND of the particular simulation shown in **a**. **c** High variability of number of GABA_B1_ immunoparticles (range 4–56) and Ca_V_2.1 immunoparticles (range 4–54) was found at the edge and inside of active zones. Box chart shows fifth, 25th, 75th, and 95th percentiles and median (bar). **d** Densities of GABA_B1_ and Ca_V_2.1 immunoparticles at the active zone and extrasynaptic sites of axon terminals. The density of both GABA_B1_ and Ca_V_2.1 immunoparticles was significantly larger at the active zones (AZ; GABA_B1_ = 301.66 ± 124.82/μm^2^; Ca_V_2.1 = 334.32 ± 130.65/μm^2^) than at extrasynaptic sites (Extra; GABA_B1_ = 45.96 ± 15.09/μm^2^; Ca_V_2.1 = 30.06 ± 20.49/μm^2^) (Kruskal–Wallis test, pairwise Mann–Whitney *U* test and Dunn’s method, *p < 0.001)

Next, we examined the co-clustering and spatial relation of GABA_B1_ and Ca_V_2.1 channels. Immunoparticles for Ca_V_2.1 were always concentrated in the presynaptic active zone of axon terminals (Fig. 9a, b), as also described previously (Kulik et al. 2004; Indriati et al. 2013). Within active zones, immunoparticles for both GABA_B1_ and Ca_V_2.1 were not homogenously distributed, but small aggregations were detected (Fig. 9a–f). To quantify the spatial relation of the two proteins, we measured the NNDs from Ca_V_2.1 (5 nm) to GABA_B1_ (10 nm) immunoparticles in active zones, and compared it with that of fitted simulations of Ca_V_2.1 particles (Fig. 10a, b). Although the mean NND (42 nm) in active zone was similar to that in dendritic shaft (44 nm), where Ca_V_2.1 and GABA_B1_ immunoparticles associated more closely than chance, we found no difference between real and simulated NNDs in active zones (Table 2, *p* = 0.77), indicating absence of significant co-clustering of GABA_B1_ with Ca_V_2.1 as a whole. On the level of individual active zone profiles, however, we found that 14% showed a significant association while only 2.9% (about chance level which is 2.5%) showed a significant dissociation. This may indicate plastic or dynamic association of GABA_B1_ with Ca_V_2.1. Interestingly, inter-cluster distance of Ca_V_2.1 and GABA_B1_ clusters in active zones was significantly smaller than in both dendritic shafts and spines (Table 3). The number of particles was highly variable and ranged from 4 to 41 per active zone for GABA_B1_ (Fig. 10c; mean = 16.3, median = 14, interquartile range = 11–21, *n* = 33 active zone profiles from three animals), and ranged from 4 to 49 per active zone for Ca_V_2.1 (Fig. 10c; mean = 19.3, median = 17, interquartile range = 12–22.2, *n* = 33 active zones three animals). Around 80% of clusters were in the range of 3–11 immunoparticles for GABA_B1_ and 3–12 immunoparticles for Ca_V_2.1, indicating that the size of clusters was similar between the receptor and the ion channel. The density of GABA_B1_ and Ca_V_2.1 at the active zones was 175.64 ± 51.07 and 200.35 ± 42.77 immunogold/μm^2^, respectively (Fig. 10d). Low density of both GABA_B1_ (mean = 45.96 immunogold/μm^2^, median = 40.8, interquartile range = 35.7–53.6, *n* = 15 profiles from three animals) and Ca_V_2.1 (mean = 19.68 immunogold/μm^2^, median = 17.4, interquartile range = 13–21.9, *n* = 15 profiles from three animals) were detected outside the active zone, along the extrasynaptic plasma membrane of axon terminals (Fig. 10d).

## Discussion

The present study describes the two-dimensional distribution of the GABA_B1_ subunit of the GABA_B_ receptor and its spatial relationship to the GIRK2 subunit of the GIRK channel and the Ca_V_2.1 subunit of P/Q-type Ca^2+^ channels along the plasma membrane of PCs using the highly sensitive and quantitative immunogold SDS-FRL technique. Our data reveal novel insights into the subcellular localization of GABA_B_ receptors and the selective coupling of these receptors to effector ion channels. Notably, we have demonstrated that although GABA_B1_ receptors are distributed along the entire somato-dendritic domain, they are not homogeneously distributed, instead showing a distance-dependent localization along the cell surface of PCs. Moreover, our data suggest that the GABA_B_–GIRK channel and GABA_B_–Ca_V_2.1 signalling cascades are present along the dendritic domains of PCs, while showing compartment-specific differences. Indeed, double-labelling studies revealed that GABA_B1_ and GIRK2 showed a high degree of co-clustering on dendritic spines, whereas GABA_B1_ and Ca_V_2.1 were mainly co-clustering on dendritic shafts. Finally, our data highlight some differences in the presynaptic co-localization of GABA_B_ receptors with their effector ion channels between distinct domains of the axon terminals.

### Distance-dependent increase of GABA_B_ receptors in PC dendrites

GABA_B_ receptors are highly expressed in the cerebellar cortex, and are found at a particularly high density in PCs, consistent with previous in situ hybridization (Kaupmann et al. 1998; Bischoff et al. 1999; Liang et al. 2000) and immunohistochemical (Fritschy et al. 1999; Kulik et al. 2002; Luján and Shigemoto 2006) studies. Previous pre-embedding immunogold data showed that the highest densities of the two GABA_B_ receptor subunits, GABA_B1_ and GABA_B2_, are found in PC spines (Kulik et al. 2002; Luján and Shigemoto 2006). Our data obtained using more sensitive approaches show the highest density of GABA_B1_ immunolabelling in dendritic compartments, particularly around the glutamatergic synapses between PC spines and parallel fibre axon terminals. These results correlate with electrophysiological data showing that GABA_B_ receptors are responsible for mediating major cellular functions in PCs. For example, PCs are hyperpolarized following GABA_B_ receptor activation (Vigot and Batini 1997), and GABA_B_ receptor activation suppresses the rebound potentiation of inhibitory synaptic inputs to PCs (Kawaguchi and Hirano 2000). Moreover, GABA_B_ receptor activation enhances postsynaptic responses mediated by mGlu_1_ receptors (Hirono et al. 2001), while also enhancing long-term depression of a glutamate-evoked currents, increasing the magnitude of depression (Kamikubo et al. 2007).

While GABA_B_ receptor immunoparticles were present along the entire somato-dendritic axis of PCs, they were distributed in non-uniform fashion. The density of GABA_B1_ immunoparticles increased from the soma towards dendritic spines, with significant differences observed between the main apical dendrites, oblique dendrites, or dendritic spines at approximately the same distance from the soma. Although understanding how integration of signals in PC dendrites is affected by this uneven distribution of GABA_B_ receptors will require detailed electrophysiological investigations, they could contribute to the activity of the cerebellar microcircuit by amplifying and modifying synaptic signals (Hanson and Smith 2002) or synaptic plasticity (Kohl and Paulsen 2010). In other cell types, we previously reported an exclusive gradient along the somato-dendritic domains for different ion channels, including GIRK and SK channels, in hippocampal pyramidal cells (Fernández-Alacid et al. 2011; Ballesteros-Merino et al. 2012, 2014).

Regardless of the neuronal compartment, our high-resolution immunoelectron microscopic studies revealed two distinct patterns of GABA_B1_ along the surface of PCs, one consisting of GABA_B1_ isolated and other consisting of GABA_B1_ clustered in plasma membrane domains. The formation of clustered and scattered pools of GABA_B_ receptors were also observed in the neuronal plasma membrane of hippocampal pyramidal cells (Kulik et al. 2006; Degro et al. 2015), and as such, this may represent a common organizational principle in different central neurons. Similar distribution patterns have been detected for other receptors and ion channels in central neurons (Kaufmann et al. 2009, 2010; Ballesteros-Merino et al. 2012; Indriati et al. 2013). Most GABA_B1_ immunoparticles were found forming clusters and this might represent an effective way by which GABA_B_ receptors induce local changes in the electrophysiological properties of the neuronal membrane. We further analysed the size of clusters along the dendritic surface of PCs and found that the area of the clusters, the number of GABA_B1_ and density of GABA_B1_ within the clusters were similar among the different dendritic compartments. These data indicate that the somato-dendritic gradient observed in PCs is due to a progressive increase in the number of clusters rather than an increase in the size and/or composition of GABA_B1_ clusters.

### Compartment-dependent co-clustering of postsynaptic GABA_B_ receptors with ion channels

One of the best-characterised downstream effectors modulated by GABA_B_ receptors is the GIRK channel (Lüscher et al. 1997). Activation of postsynaptic GABA_B_ receptors generally causes activation of GIRK channels, thereby hyperpolarizing the postsynaptic plasma membrane (Kaupmann et al. 1998). In the cerebellum, GIRK channels are expressed in a cell type-dependent manner (Aguado et al. 2008), and PCs express GIRK1, GIRK2, and GIRK3 (Karschin et al. 1996). We have demonstrated that GIRK1, GIRK2, and GIRK3 subunits co-precipitated together in the cerebellum (Aguado et al. 2008), and further revealed that GIRK subunits co-immunoprecipitated GABA_B_ receptors in an expression system and solubilised cerebellar membranes (Fernández-Alacid et al. 2009; Ciruela et al. 2010). The association GIRK/GABA_B_ is not supported by proteomic approaches (Schwenk et al. 2016), possibly suggesting that the interaction is via G protein. Furthermore, GABA_B_ receptors preferentially localise to the extrasynaptic plasma membrane of PC spines, where they co-localise with GIRK channels (Fernández-Alacid et al. 2009). Consistent with these data, our immunogold labelling revealed a high degree of co-clustering of GABA_B1_ and GIRK2 on dendritic spines, whereas on dendritic shafts they are mainly segregated. Similar spine-specific co-clustering of GABA_B1_ and GIRK3 has been found in hippocampal pyramidal cells (Kulik et al. 2006). In spines, the mean distance between the ion channels to the receptor was 43 nm, and this short distance suggests the existence of preformed macromolecular complexes that would ensure reliable and efficient GABA_B_–GIRK interaction (Ciruela et al. 2010). However, the observed segregation between GABA_B_ receptors and GIRK channels on dendritic shafts raises the question as to how GIRK channels are activated in this compartment. GIRK channels may be activated by GABA_B_ receptors, as the mean distance between the receptor and ion channel in dendritic shafts (143 nm) may be sufficient for GABA_B_–GIRK coupling (Karschin 1999). It is also possible that GIRK channels couple to a different GPCR(s), including metabotropic glutamate receptors, adenosine A1 receptors, cannabinoid CB2 receptors; indeed, these receptors have been linked to GIRK channel activation in the cerebellum (North 1989).

If GABA_B_ receptors do not couple to GIRK channels in dendritic shafts, N- or P/Q-type voltage-dependent Ca^2+^ channels might (Bettler et al. 2004). Although GABA_B_ receptors couple with Ca_V_2.1 channels at presynaptic sites (Huston et al. 1995; Takahashi et al. 1998), there is evidence indicating that they also trigger Ca^2+^ influx across postsynaptic membranes (Catterall 1998). Light and electron microscopic studies have shown that PCs also express high density of Ca_V_2.1 channels (Kulik et al. 2004; Indriati et al. 2013). Therefore, it seems reasonable to consider that GABA_B_ receptors and Ca_V_2.1 channels are functionally coupled at postsynaptic compartments. Supporting this idea, we demonstrated that Ca_V_2.1 co-immunoprecipitated with GABA_B_ receptors in solubilised cerebellar membranes. This association between Ca_V_2.1 and GABA_B_ may be indirect via KCTD16 (Schwenk et al. 2016; Pin and Bettler 2016). However, the neuronal compartments where these effectors couple to GABA_B_ receptors in PCs should be further elucidated.

Similar to the observed distribution of GABA_B_ receptors, Ca_V_2.1 channels are distributed non-uniformly from soma to distal dendrite with graded increase in density (Indriati et al. 2013). The present study shows that receptor and ion channel distributions are overlapping, but show divergence in their co-clustering pattern. We found that the mean nearest distance of Ca_V_2.1 to GABA_B1_ was 44 nm in dendritic shafts, and about twice that distance (82.2 nm) in spines. Therefore, our study confirms that GABA_B_–Ca_V_2.1 complexes are present along the dendritic domains of cerebellar PCs, and suggest a preferential and more efficient coupling in dendritic shafts. This coupling is supported by the results of whole-cell patch recording showing that GABA_B_ receptors inhibit P-type Ca^2+^ channels through a G protein-mediated mechanism (Mintz and Bean 1993). Regardless of the possible coupling between GABA_B_ receptors and Ca_V_2.1 channels in dendritic spines, their close spatial relationship was not unexpected. First, activation of GABA_B_ receptors by agonists or extracellular Ca^2+^ enhanced mGlu_1_-mediated inward currents and Ca^2+^ signals in PCs, demonstrating a cross-talk between mGlu_1_ and GABA_B_ receptors (Hirono et al. 2001; Tabata et al. 2004). Second, previous studies showed a direct molecular and functional coupling between Ca_V_2.1 channels and the mGlu_1_ receptor (Kitano et al. 2003). Therefore, it seems reasonable to expect that Ca_V_2.1 channels distributed in dendritic spines might also be involved in spatiotemporal regulation of intracellular Ca^2+^ in glutamatergic neurotransmission through both mGlu and GABA_B_ receptors.

### Short distance of GABA_B_ receptors from ion channels in axon terminals

The release of neurotransmitter can be modulated by GABA_B_ receptors inhibiting the action of Ca_V_2.1 and Ca_V_2.2 channels (Mintz and Bean 1993; North 1989; Lüscher et al. 1997; Takahashi et al. 1998). We observed a high density of immunoparticles for Ca_V_2.1 in a restricted area of the presynaptic plasma membrane, suggesting a preferential localization of Ca_V_2.1 channels at the active zone of axon terminals, as described (Indriati et al. 2013). Indeed, double‐labelling for Ca_V_2.1 and presynaptic active zone proteins RIM1 and RIM2 provided evidence that these proteins were confined to the same compartment of the presynaptic plasma membrane in the cerebellum (Baur et al. 2015). Although our quantitative analysis showed that Ca_V_2.1 was not significantly associated to GABA_B_ receptor compared with simulated Ca_V_2.1 in the active zone, the mean NND from Ca_V_2.1 to GABA_B_ particles (42 nm) was comparable to that in dendritic shafts (44 nm), where they significantly co-clustered. This is because of the high overall density of both molecules in the active zone, which may allow close interaction between GABA_B_ receptor and Ca_V_2.1 channels in the presynaptic active zone.

We previously reported that the three GIRK channel subunits are localised at presynaptic sites in the cerebellum (Aguado et al. 2008; Fernández-Alacid et al. 2009), as well as in other brain regions (Morishige et al. 1996; Ponce et al. 1996; Koyrakh et al. 2005; Marker et al. 2005). Although electrophysiological studies do not support a role for a pre-synaptic GIRK activation as a primary mechanism by which GABA_B_ receptors modulate neurotransmitter release (Lüscher et al. 1997), using functional assays we reported that GIRK channel-mediated inhibition of glutamate release occurs through GABA_B_ receptors in the cerebral cortex (Ladera et al. 2008) and cerebellum (Fernández-Alacid et al. 2009). The presynaptic coupling in the cerebellum is supported by the up-regulation of GIRK3 and GABA_B_ receptors in parallel fibre terminals after genetic ablation of GABA_B1_ and GIRK3, respectively (Fernández-Alacid et al. 2009). Using more sensitive immunolocalisation techniques, we not only confirmed the presynaptic distribution of GIRK channels but also revealed short NND (42 nm) to GABA_B_ receptors, which is exactly the same NND as in spine (42 nm). Although we did not find a significant difference between real and simulated inter-NNDs, their proximity to each other suggests an involvement of GABA_B_–GIRK interaction in the regulation of neurotransmitter release (Ladera et al. 2008; Fernández-Alacid et al. 2009). Altogether, our data clearly suggest that coupling of GABA_B_ receptors to their effector ion channels differs in dendritic spine and shaft domains but may be similar in axon terminal domains.

Altogether, our data clearly demonstrated that both particle and the shortest particle and cluster NNDs for Ca_V_2.1 to GABA_B_ and GIRK to GABAB occurred in the dendritic shaft and dendritic spine, respectively, consistent with the functional associations between the ion channels and the receptor in the respective compartments. The cluster NNDs for Ca_V_2.1/GIRK to GABA_B_ in the active zone were also shorter than those observed in dendrites and spines, suggesting that similar molecular and functional interaction can take place in the active zone despite of no significant difference from the simulated distribution.
